# Association Between Severe Nonadherence to Hydroxychloroquine and Systemic Lupus Erythematosus Flares, Damage, and Mortality in 660 Patients From the SLICC Inception Cohort

**DOI:** 10.1002/art.42645

**Published:** 2023-11-13

**Authors:** Yann Nguyen, Benoît Blanchet, Murray B. Urowitz, John G. Hanly, Caroline Gordon, Sang‐Cheol Bae, Juanita Romero‐Diaz, Jorge Sanchez‐Guerrero, Ann E. Clarke, Sasha Bernatsky, Daniel J. Wallace, David A. Isenberg, Anisur Rahman, Joan T. Merrill, Paul R. Fortin, Dafna D. Gladman, Ian N. Bruce, Michelle Petri, Ellen M. Ginzler, Mary Anne Dooley, Rosalind Ramsey‐Goldman, Susan Manzi, Andreas Jönsen, Graciela S. Alarcón, Ronald F. Van Vollenhoven, Cynthia Aranow, Véronique Le Guern, Meggan Mackay, Guillermo Ruiz‐Irastorza, S. Sam Lim, Murat Inanc, Kenneth C. Kalunian, Søren Jacobsen, Christine A. Peschken, Diane L. Kamen, Anca Askanase, Jill Buyon, Nathalie Costedoat‐Chalumeau

**Affiliations:** ^1^ National Referral Centre for Rare Autoimmune and Systemic Diseases, Hôpital Cochin, AP‐HP Centre and Université Paris Cité and Centre de Recherche en Epidémiologie et Statistiques (CRESS), Unité Inserm 1153, Université de Paris Cité Paris France; ^2^ Biologie du médicament‐Toxicologie, AP‐HP Centre–Hôpital Cochin, Université Paris Cité, and UMR8038 CNRS, U1268 INSERM, Université Paris Cité, PRES Sorbonne Paris Cité, CARPEM Paris France; ^3^ Toronto Western Hospital, University of Toronto Toronto Ontario Canada; ^4^ Queen Elizabeth II Health Sciences Centre and Dalhousie University Halifax Nova Scotia Canada; ^5^ Institute of Inflammation and Ageing, University of Birmingham Birmingham United Kingdom; ^6^ Hanyang University Hospital for Rheumatic Diseases, Hanyang University Institute for Rheumatology, and Hanyang University Institute of Bioscience and Biotechnology Seoul Korea; ^7^ Instituto Nacional de Ciencias Médicas y Nutrición Mexico City Mexico; ^8^ Mount Sinai Hospital and University Health Network, University of Toronto Toronto Canada; ^9^ Cumming School of Medicine University of Calgary Calgary Alberta Canada; ^10^ McGill University Health Centre Quebec Canada; ^11^ University of California Los Angeles; ^12^ University College London UK; ^13^ Oklahoma Medical Research Foundation Oklahoma City; ^14^ CHU de Québec–Université Laval Québec City Canada; ^15^ NIHR Manchester Biomedical Research Centre, Manchester University Hospitals NHS Foundation Trust, Manchester Academic Health Science Center and Centre for Epidemiology Versus Arthritis, The University of Manchester Manchester UK; ^16^ Johns Hopkins University School of Medicine Baltimore Maryland; ^17^ State University of New York Downstate Medical Center Brooklyn; ^18^ Thurston Arthritis Research Center, University of North Carolina Chapel Hill; ^19^ Northwestern University and Feinberg School of Medicine Chicago Illinois; ^20^ Allegheny Health Network Pittsburgh Pennsylvania; ^21^ Lund University Lund Sweden; ^22^ University of Alabama at Birmingham Marnix E. Heersink School of Medicine; ^23^ University of Amsterdam Amsterdam Noord‐Holland The Netherlands; ^24^ Feinstein Institute for Medical Research Manhasset New York; ^25^ National Referral Centre for Rare Autoimmune and Systemic Diseases, Hôpital Cochin, AP‐HP Centre, Université Paris Cité Paris France; ^26^ BioCruces Bizkaia Health Research Institute, University of the Basque Country Barakaldo Spain; ^27^ Emory University School of Medicine Atlanta Georgia; ^28^ Istanbul University Istanbul Turkey; ^29^ University of California San Diego School of Medicine La Jolla; ^30^ Rigshospitalet, Copenhagen University Hospital Copenhagen Denmark; ^31^ University of Manitoba Winnipeg Manitoba Canada; ^32^ Medical University of South Carolina Charleston; ^33^ Hospital for Joint Diseases and, Seligman Centre for Advanced Therapeutics, New York University New York City; ^34^ New York University School of Medicine New York City

## Abstract

**Objective:**

The goals of this study were to assess the associations of severe nonadherence to hydroxychloroquine (HCQ), objectively assessed by HCQ serum levels, and risks of systemic lupus erythematosus (SLE) flares, damage, and mortality rates over five years of follow‐up.

**Methods:**

The Systemic Lupus International Collaborating Clinics (SLICC) Inception Cohort is an international multicenter initiative (33 centers throughout 11 countries). The serum of patients prescribed HCQ for at least three months at enrollment were analyzed. Severe nonadherence was defined by a serum HCQ level <106 ng/mL or <53 ng/mL for HCQ doses of 400 or 200 mg/day, respectively. Associations with the risk of a flare (defined as a Systemic Lupus Erythematosus Disease Activity Index 2000 increase ≥4 points, initiation of prednisone or immunosuppressive drugs, or new renal involvement) were studied with logistic regression, and associations with damage (first SLICC/American College of Rheumatology Damage Index [SDI] increase ≥1 point) and mortality with separate Cox proportional hazard models.

**Results:**

Of the 1,849 cohort participants, 660 patients (88% women) were included. Median (interquartile range) serum HCQ was 388 ng/mL (244–566); 48 patients (7.3%) had severe HCQ nonadherence. No covariates were clearly associated with severe nonadherence, which was, however, independently associated with both flare (odds ratio 3.38; 95% confidence interval [CI] 1.80–6.42) and an increase in the SDI within each of the first three years (hazard ratio [HR] 1.92 at three years; 95% CI 1.05–3.50). Eleven patients died within five years, including 3 with severe nonadherence (crude HR 5.41; 95% CI 1.43–20.39).

**Conclusion:**

Severe nonadherence was independently associated with the risks of an SLE flare in the following year, early damage, and five‐year mortality.

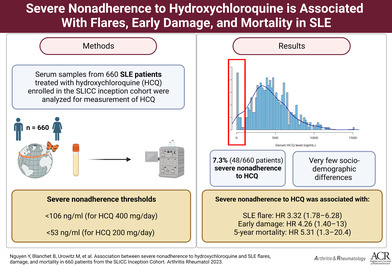

## INTRODUCTION

Systemic lupus erythematosus (SLE) is a multisystem autoimmune disease in which preventing adverse long‐term outcomes remains a major challenge. The efficacy of antimalarials, especially hydroxychloroquine (HCQ), is well established.^1^, ^2^ Besides reducing the risk of SLE flares, HCQ is beneficial against SLE‐related comorbidities, including diabetes, thrombotic events, and dyslipidemia,^3^, ^4^, ^5^, ^6^, ^7^ and against long‐term damage^8^, ^9^, ^10^ and death.^11^ It is widely recommended that patients with SLE receive this treatment.^12^, ^13^, ^14^

Like all self‐administered medications, HCQ's effectiveness is impaired by nonadherence, reported to range between 3% and 85% in SLE.^8^, ^15^, ^16^, ^17^, ^18^, ^19^, ^20^, ^21^, ^22^, ^23^, ^24^, ^25^, ^26^, ^27^, ^28^, ^29^, ^30^ Low whole blood HCQ levels are a marker of SLE exacerbation because of their pharmacokinetic/pharmacodynamic relations.^16^ Because its blood half‐life is at least 5 days, and its terminal half‐life 43 days,^31^ a very low blood HCQ level is an objective indicator of severe nonadherence, identifying patients who have not taken HCQ for a significant period of time and not those who have just missed a few tablets.^16^, ^17^, ^25^, ^26^, ^27^, ^28^, ^30^, ^32^, ^33^, ^34^ In a first study, published in 2007, we retrospectively validated an HCQ cutoff <200 ng/ml in whole blood to identify severely nonadherent patients.^16^ Other cutoffs have been proposed since then: 500 ng/ml,^35^ 100 ng/ml, 15 ng/ml,^17^ or undetectable whole‐blood HCQ levels. Most studies have measured HCQ in whole blood. Large longitudinal cohorts, however, most often collect serum samples, with whole blood samples being relatively rare. Recently, we compared whole blood and serum levels^36^ and found a mean serum/whole blood HCQ ratio of 0.53 ± 0.15. We concluded that when whole blood is unavailable, serum HCQ levels can be used to assess nonadherence.

In this study, we aimed to assess whether patients prescribed HCQ for at least three months but with objective severe nonadherence, defined by very low HCQ serum levels, were at higher risk of SLE flares in the subsequent year, and of damage and death up to five years later.

**What is already known on this topic**

- In addition to reducing the risk of systemic lupus erythematosus (SLE) flares, hydroxychloroquine has multiple benefits against SLE‐related comorbidities and the risks of long‐term damage and of mortality.
- Hydroxychloroquine's effectiveness is impaired by nonadherence, reported to range from 3% to 85% in patients with SLE.

**What this study adds**

- In the large, international, multicenter, longitudinal Systemic Lupus International Collaborating Clinics cohort, using serum hydroxychloroquine levels, we found a 7.3% rate of severe nonadherence.
- Severe nonadherence to hydroxychloroquine was independently associated with the risk of an SLE flare, early damage, and mortality.

**How this study might affect research, practice, or policy**

- Our results suggest the benefits of testing for detecting severe nonadherence and of dedicating more resources and more time to these patients to improve their long‐term prognosis.

## PATIENTS AND METHODS

### The SLICC Inception Cohort

The Systemic Lupus International Collaborating Clinics (SLICC) Inception Cohort was recruited between 1999 and 2011 from 33 centers in 11 countries within North America, Europe, and Asia.^8^, ^37^ Patients were enrolled within 15 months of fulfilling at least four of the 1997 American College of Rheumatology (ACR) revised classification criteria for SLE.^38^ After the enrollment visit, patients were seen annually at their study center by a clinician, who completed a detailed case report form. Data were submitted to the coordinating center at the University of Toronto for storage in a centralized database. Annual serum samples have been collected from most patients.

### Study participants

We analyzed serum samples of patients who were prescribed HCQ for at least three months at cohort enrollment. The current HCQ course, including its start date and its average dose, were collected at enrollment and at each subsequent visit. We used sera sampled at enrollment in the cohort, or, if unavailable, during the first‐year follow‐up visit after enrollment. The date of the serum sample corresponded to time zero (T0). Patients not treated with HCQ (ie, those for whom the drug was contraindicated), treated for less than three months at enrollment, or who had no follow‐up visit after T0 were excluded. The Institutional Research Ethics Boards of participating centers approved the SLICC Inception Cohort Study in accordance with the Declaration of Helsinki guidelines for research in humans. All patients provided written informed consent.

### Serum hydroxychloroquine measurement and definition of severe nonadherence

All serum HCQ levels were assayed at Cochin Hospital by a previously published method.^41^ In this study, we compared whole blood and serum levels^36^ and found mean ± SD HCQ concentrations of 469 ± 223 ng/mL in serum and 916 ± 449 ng/mL in whole blood, for a mean serum:whole blood HCQ ratio of 0.53 ± 0.15. Two independent groups subsequently confirmed this result, reporting ratios of 0.51^37^ and 0.54^41^ and high reproducibility. To determine if serum HCQ level cutoffs could be established to identify severely nonadherent patients, we calculated the following thresholds for nonadherence by extrapolation: prescribed HCQ dose of 400 mg/day: <106 ng/mL (corresponding to 200 ng/mL in whole blood); prescribed HCQ dose of 200 mg/day: <53 ng/ml (100 ng/mL in whole blood); other prescribed HCQ daily doses were rounded to the nearest of 200 or 400 mg/day. The 300 mg/day dose was rounded up to 400 mg/day.

In our previous studies, relatively few patients took 200 mg/day (7%–15%),^16^, ^30^ and we used the same cutoff for patients treated with 200 and 400 mg/day. The relation between HCQ daily dose and HCQ blood level is nonetheless linear, as shown in 2016,^25^ and patients treated with 200 mg/day are expected to have blood or serum HCQ levels half those of patients treated with 400 mg/day. We thus chose to adapt our thresholds with different thresholds based on the daily HCQ dose. We also, however, conducted two sensitivity analyses that defined severe nonadherence in all patients by the thresholds of 106 ng/mL and 53 ng/mL, regardless of daily HCQ dose. Patients were considered to have nonquantifiable serum HCQ levels at <20 ng/mL (lower limit of quantification).

### Clinical variables

Data from T0 included the following: demographic features including age, sex, Black race (yes/no), educational level (high school education or less vs postsecondary education), dyslipidemia, diabetes mellitus, and body mass index (BMI; <18; 18–25; 25–30, >30 kg/m^2^). We collected current prescriptions of prednisone and other immunosuppressive medications (methotrexate, azathioprine, mycophenolate mofetil, rituximab, and oral or intravenous [IV] cyclophosphamide) at T0, again including their start dates.

Follow‐up data collected over the subsequent five years included SLE activity, defined by the Systemic Lupus Erythematosus Disease Activity Index 2000 (SLEDAI‐2K),^42^ damage defined by the SLICC/American College of Rheumatology Damage Index (SDI),^43^, ^44^ new (since the previous visit) course of oral or IV prednisone or other immunosuppressive agent with its start date, any new renal involvement since the previous visit, and deaths. Causes of death were collected and analyzed.

### Outcome definition

An SLE flare was defined by a composite outcome involving at least one of the following events in the first year after T0: (a) increase of at least 4 points in the SLEDAI‐2K; (b) new start of prednisone (oral or IV) or other immunosuppressive agent (azathioprine, methotrexate, mycophenolate mofetil, rituximab, oral or IV cyclophosphamide); (c) new renal involvement since the last visit, including new active nephritis, defined by hematuria (>5 red blood cells/high power field [HPF]) and/or pyuria (>5 white blood cells/HPF), both after exclusion of other causes, a new or recent increase of >500 mg 24‐hour protein, or heme granular or red blood cell casts; or new nephrotic syndrome. Increased damage was defined by an SDI increase of ≥1 point in the five years after T0.

### Statistical analyses

Descriptive statistics were used to summarize enrollment data: counts (percentages) for categorical variables and medians (interquartile ranges) or mean ± SD for continuous variables. Characteristics of patients at T0 with and without severe HCQ nonadherence were compared by Student's *t*‐tests for continuous variables and chi‐squared tests for categorical variables.

To assess the association between severe nonadherence and an SLE flare, we used logistic regression models, with and without adjustment for potentially relevant variables assessed at T0. Sensitivity analyses studied each individual outcome of the primary endpoint separately and assessed the association between nonquantifiable serum HCQ levels and the primary composite outcome. We also performed two other sensitivity analyses, one applying a severe nonadherence threshold of 106 ng/mL and the other 53 ng/mL, for all patients, regardless of their prescribed daily dose.

The association between severe nonadherence and the risk of increased damage was assessed by survival analysis. For five years after T0, patients contributed person‐time from T0 until the first worsening score, loss to follow‐up, or death, whichever occurred first. To assess the risk of early damage, we computed sensitivity survival analyses censoring patients at one, two, three, and four years. Patients with no available SDI score recorded at T0 (because they had been diagnosed for less than six months) had it imputed by the SDI value at the first follow‐up visit. Associations were assessed with Cox proportional hazard models, adjusted for sex, educational level, and relevant covariables associated with SDI worsening in univariate Cox models. Among patients with an SDI increase of ≥1 point in the five years after HCQ measurement, damage included in the SDI was compared between nonadherent patients and the others. We also separately compared damage that was more likely to be related to steroid/cyclophosphamide treatment (including cataracts, retinal change, or optic atrophy, muscle atrophy or weakness, osteoporosis, premature gonadal failure, and diabetes mellitus) versus other damage considered related to SLE itself.

Finally, we assessed the association between severe nonadherence and deaths (all causes) in the five years after T0 with Cox proportional hazard models. All analyses were performed with R version 3.6.1 (R Foundation for Statistical Computing). Data are available upon reasonable request.

## RESULTS

### Study population

By February 2021, of the 1,849 patients enrolled in the SLICC Inception Cohort, 824 had been treated with HCQ for three months or longer at enrollment (Supplementary Figure 1). Among these 824 patients, 663 had an available serum sample from the enrollment visit or during the first follow‐up visit and met the inclusion criteria. Serum HCQ levels were measured for 660 (99.5%) patients; the other three had technical issues (insufficient serum quantity). Compared with the excluded patients, our study population was slightly older (34 vs 31 years, *P* = 0.002) and more likely to have a postsecondary education level (63% vs 55%, *P* < 0.001), but had a lower frequency of renal (20% vs 32%, *P* < 0.001) and neurologic involvement (2.7% vs 6%, *P* = 0.002) (Supplementary Table 1). The interval between their diagnosis and inclusion was longer (6.8 vs 4.0 months, *P* < 0.001), because patients taking HCQ for less than three months at enrollment were excluded. The populations did not differ for sex, cigarette smoking, or Black race.

The HCQ samples were taken at T0, which was either cohort enrollment for 634 (96%), or the first follow‐up visit for 26 (3.9%). Table 1 presents patients’ characteristics at T0. Median follow‐up was 6.1 years (interquartile range [IQR] 3.0–9.7 years) after T0, and 401 (61%) patients were followed up for at least five years.

**Table 1:** Characteristics of the study population and outcomes, according to severe nonadherence to HCQ at T0*

Patients’ characteristics	Overall (n = 660)	Severe nonadherence to HCQ		*P* value
		No (n = 612)	Yes (n = 48)	
Female sex, n (%)	580 (87.9)	536 (87.6)	44 (91.7)	0.545
Pregnancy, n (%)	7 (1.1)	6 (1.0)	1 (2.1)	1.00
Black race, n (%)	107 (16.2)	96 (15.7)	11 (22.9)	0.269
Age at serum sample, mean ± SD, y	36.2 ± 13.5	36.4 ± 13.7	33.4 ± 10.7	0.132
Months since SLE diagnosis, mean ± SD	7.2 ± 4.6	7.1 ± 4.6	8.4 ± 4.9	0.062
Education level, n (%)				0.886
Postsecondary	413 (62.6)	382 (62.4)	31 (64.6)	
High school or less	247 (37.4)	230 (37.6)	17 (35.4)	
Cigarette smoking, n (%)				0.642
Nonsmoker	437 (66.2)	408 (66.7)	29 (60.4)	
Current or past smoker	222 (33.6)	203 (33.2)	19 (39.6)	
Not available	1 (0.2)	1 (0.2)	0 (0.0)	
Main clinical manifestations, n (%)				
Renal disease	131 (19.8)	120 (19.6)	11 (22.9)	0.715
Neurologic disorder	18 (2.7)	16 (2.6)	2 (4.2)	0.861
SLEDAI‐2K at T0, mean ± SD	4.8 ± 4.9	4.8 ± 4.8	6.0 ± 5.8	0.091
Other comorbidities				
BMI, mean ± SD, kg/m^2^	25.8 ± 6.1	25.7 ± 6.0	27.3 ± 7.2	0.074
Dyslipidemia, n (%)	55 (8.3)	50 (8.2)	5 (10.4)	0.786
Diabetes mellitus, n (%)	17 (2.6)	16 (2.6)	1 (2.1)	0.658
Treatment at T0, n (%)				
HCQ, daily dose				
200 mg/day	155 (23.5)	143 (23.4)	12 (25.0)	0.936
400 mg/day	505 (76.5)	469 (76.6)	36 (75.0)	
Corticosteroids	438 (66.4)	404 (66.0)	34 (70.8)	0.602
Other immunosuppressive drugs	242 (36.7)	219 (35.8)	23 (47.9)	0.127
Azathioprine	91 (13.8)	78 (12.7)	13 (27.1)	0.011
Cyclophosphamide	25 (3.8)	22 (3.6)	3 (6.2)	0.592
Methotrexate	65 (9.8)	62 (10.1)	3 (6.2)	0.537
Mycophenolate mofetil	58 (8.8)	53 (8.7)	5 (10.4)	0.881
Other immunosuppressant^a^	7 (1.1)	6 (1.0)	1 (2.1)	1
Outcomes, n (%)				
SLE flare within one year	191 (28.9)	163 (26.6)	28 (58.3)	<0.001
≥4‐point increase in SLEDAI‐2K	68 (10.3)	57 (9.3)	11 (22.9)	0.006
New steroid and/or IS	94 (14.2)	78 (12.7)	16 (33.3)	<0.001
New renal involvement	71 (10.8)	62 (10.1)	9 (18.8)	0.107
≥1‐point increase SDI within 5 y	167 (25.3)	152 (24.8)	15 (31.2)	0.417
Death within 5 y	11 (1.7)	8 (1.3)	3 (6.2)	0.047

* HCQ, hydroxychloroquine; IS, immunosuppressive drug; kg/m^2^, kilograms per square meter; SD, standard deviation; SDI, Systemic Lupus International Collaborating Clinics/American College of Rheumatology Damage Index; SLE, systemic lupus erythematosus; SLEDAI‐2K, Systemic Lupus Erythematosus Disease Activity Index 2000; T0, time zero.

^a^ Other immunosuppressant users included four patients treated with cyclosporine, one with sulfasalazine, and one with intravenous immunoglobulins (in the “not severely nonadherent” group), and one patient with rituximab (in the “nonadherent” group).

### HCQ levels and nonadherence at T0

HCQ had been prescribed for a mean ± SD of 8.7 ± 10.4 months before T0: 7.4 ± 5.5 months for nonadherent patients versus 8.8 ± 10.7 months for the others (*P =* 0.373). The daily HCQ dose was 400 mg for 428 patients and 200 mg for 141 patients, and the other 91 doses were rounded to the closest daily prescription; doses of 300 mg (n = 62) were rounded up to 400 mg/day. Overall, the median serum HCQ level was 388 ng/mL (IQR 244–566 ng/mL).

For the 155 patients with prescribed HCQ doses of or rounded to 200 mg/day, the median HCQ level was 250 ng/mL (IQR 158–365 ng/mL). Twelve (7.7%) had an HCQ level <53 ng/mL and were thus considered severely nonadherent. For the 505 patients with an HCQ prescription of or rounded to 400 mg/day, the median HCQ level was 427 ng/mL (IQR 287–602 ng/mL); 36 (7.1%) had an HCQ level <106 ng/mL and were thus severely nonadherent.

**Figure 1:**
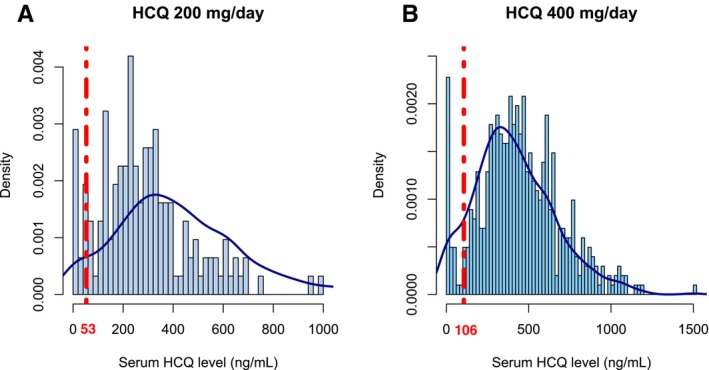
Distribution of serum hydroxychloroquine (HCQ) levels according to daily prescribed dose: (**A**) 200 mg/day and (**B**) 400 mg/day. The red dotted lines represent thresholds for severe nonadherence to HCQ: (**A**) 53 ng/mL and (**B**) 106 ng/mL.

Accordingly, the overall population contained 48 (7.3%) severely nonadherent patients, 28 (4.2% of the overall cohort) of whom had nonquantifiable serum HCQ levels. Figure 1 presents the distributions of HCQ levels in the overall population and by prescribed HCQ dose.

Of note, among the 62 patients with an HCQ prescription of 300 mg/day, 3 (4.8%) had an HCQ level <106 ng/mL. Moreover, among the seven pregnant patients at T0, only one was considered nonadherent, with an undetectable level of HCQ. None had flares or increased their SDI during follow‐up.

### Factors associated with severe nonadherence to HCQ at T0

No sociodemographic, clinical, or laboratory factors were clearly associated with severe nonadherence (Table 1). In the univariate analyses, although the current prescription of immunosuppressive treatment at T0 did not differ significantly between the groups, azathioprine treatment was more frequently currently prescribed in severely nonadherent patients at T0 (27.1% vs 12.7%; *P* = 0.011). Among the nonadherent patients, azathioprine was prescribed before or concomitantly with HCQ in 72.7% (8 of 11 patients with an available start date), and was prescribed 0.5, 0.8, and 1.2 months after HCQ for the other three patients (Supplementary Figure 2). Several other variables also showed nonsignificant trends, including higher SLEDAI‐2K at T0 (6.0 vs 4.8), higher BMI at T0, (27.3 vs 25.7 kg/m^2^), and Black race (22.9% vs 16.2%) among nonadherent patients.

### Severe nonadherence to HCQ at T0 and risk of SLE flares at 1 year

An SLE flare occurred in the year after T0 in 163 patients without and 28 patients (58.3%) with severe nonadherence. An increase of at least 4 points in the SLEDAI‐2K was observed in 57 patients(9.3%) without and 11 patients (22.9%) with severe nonadherence. New prednisone and/or another immunosuppressive drug was prescribed for 78 patients (12.7%) without and 16 patients (33.3%) with severe nonadherence, and new renal involvement was identified in 62 (10.1%) and 8 patients (18.8%), respectively (Table 1).

In the univariate analyses, age, race, SLEDAI‐2K at T0, current course of immunosuppressive treatments at T0, and severe HCQ nonadherence were all associated with the SLE flare risk (Table 2). In the multivariate analysis, severe nonadherence constituted the most important independent risk factor for flare (adjusted OR [aOR] 3.32; 95% confidence interval [CI] 1.78–6.28) (Table 2).

**Table 2:** Odds ratios (95% confidence intervals) for the risk of an SLE flare in the year after the HCQ measurement at T0*

Demographic data and comorbidities	Overall (n = 660)	SLE flare within one year^a^		
		Patients (n = 191)	Univariate OR (95% CI)	Multivariate OR (95% CI)^b^
Age at serum sample, mean ± SD, y	36.2 ± 13.5	33.3 ± 12.2	0.98 (0.96–0.99)	0.98 (0.97–0.99)
Male, n (%)	580 (87.9)	167 (87.4)	1.06 (0.63–1.75)	–
Black race, n (%)	107 (16.2)	47 (24.6)	2.22 (1.45–3.40)	2.09 (1.33–3.26)
Education level, n (%)				
Postsecondary	413 (62.6)	116 (60.7)	Reference	Reference
High school or less	247 (37.4)	75 (39.3)	1.12 (0.79–1.58)	1.10 (0.76–1.58)
Cigarette smoking, n (%)				
Nonsmoker	437 (66.2)	127 (66.8)	Reference	
Current or past smoker	222 (33.6)	63 (33.2)	0.97 (0.67–1.38)	
BMI, mean ± SD, kg/m^2^	25.8 ± 6.1	25.9 ± 6.0	1.00 (0.98–1.03)	
SLEDAI‐2K at T0, mean ± SD	4.8 ± 4.9	5.9 ± 5.7	1.06 (1.02–1.10)	1.05 (1.01–1.09)
Corticosteroids, n (%)	438 (66.4)	137 (71.7)	1.42 (0.99–2.06)	1.00 (0.67–1.51)
Azathioprine, n (%)	91 (13.8)	38 (19.9)	1.95 (1.23–3.07)	1.63 (0.99–1.51)
HCQ adherence, n (%)				
Severe HCQ nonadherence	48 (7.3)	28 (14.7)	3.86 (2.12–7.12)	3.32 (1.78–6.28)
Nonquantifiable	28 (4.2)	16 (8.4)	3.48 (1.62–7.67)	

* BMI, body mass index; CI, confidence interval; HCQ, hydroxychloroquine; IV, intravenous; OR, odds ratio; SLEDAI‐2K, Systemic Lupus Erythematosus Disease Activity Index 2000; T0, time zero.

^a^ An SLE flare was defined by occurrence of one of the following events in the first year after the T0 visit: (a) ≥4 points in the SLEDAI‐2K; (b) new start in prednisone (oral or pulsed) or other immunosuppressive agent (azathioprine, methotrexate, mycophenolate mofetil, rituximab, oral or IV cyclophosphamide); and/or (c) new renal involvement including active nephritis, new nephrotic syndrome, new dialysis, or kidney transplantation.

^b^ The following variables were included in the multivariate model: age, Black race, education level (postsecondary; high school or less), SLEDAI‐2000, corticosteroids, azathioprine, and severe HCQ nonadherence. No interaction was found between nonadherence to HCQ and age (*P* = 0.51), education (*P* = 0.47), Black race (*P* = 0.26), SLEDAI‐2K (*P* = 0.59), azathioprine (*P* = 0.23), or with corticosteroids (*P* = 0.49).

When we considered each component of the primary endpoint separately, severe HCQ nonadherence was associated with a SLEDAI‐2K increase of 4 points or more (aOR 3.19; 95% CI 1.42–6.81) (Supplementary Table 2) and with the risk of a new prednisone and/or other immunosuppressive prescription (aOR 3.16; 95% CI 1.59–6.07) (Supplementary Table 3), but not with new renal involvement (aOR 1.41; 95% CI 0.56–3.25) (Supplementary Table 4). Nonquantifiable serum HCQ levels also predicted flare risk defined by the primary composite endpoint (aOR 2.82; 95% CI 1.24–6.54, data not shown).

Finally, applying each of the two thresholds for the definition of severe nonadherence (106 ng/mL and 53 ng/mL) to all patients, regardless of the prescribed HCQ dosage, yielded similar results: these definitions were again associated with the flare risk (aOR 2.38; 95% CI 1.34–4.22 and aOR 3.01; 95% CI 1.55–5.94, respectively) (Supplementary Table 5).

### Severe nonadherence to HCQ at T0 and risk of damage at five years

**Figure 2:**
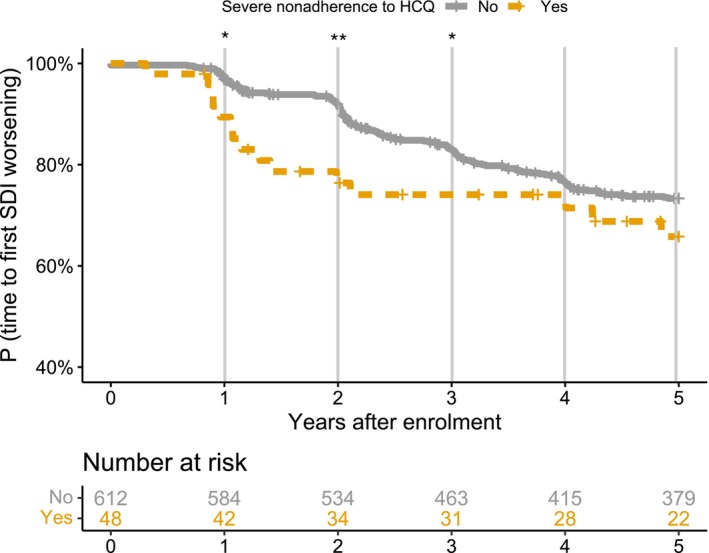
Kaplan‐Meier curves for the risk of damage, defined by a ≥1‐point increase of SDI, according to severe nonadherence. Severe nonadherence was associated with the risk of SDI worsening at one (adjusted HR 4.26; 95% CI 1.40–13), two (adjusted HR 3.54; 95% CI 1.83–6.86), and three years after the HCQ measurement (adjusted HR 1.92; 95% CI 1.05–3.50), with a nonsignificant trend at five years (adjusted HR 1.47; 95% CI 0.86–2.49). The following variables were included in the multivariate models: age, Black race, education level (postsecondary; high school or less), SLEDAI‐2K, azathioprine, and severe HCQ nonadherence. CI, 95% confidence interval; HCQ, hydroxychloroquine; HR, hazard ratio; SDI, Systemic Lupus International Collaborating Clinics/American College of Rheumatology Damage Index; SLEDAI‐2K, Systemic Lupus Erythematosus Disease Activity Index 2000. *P < 0.05; **P < 0.01; NS, nonsignificant.

In the five years after T0, the SDI of 167 patients (25.3%) had increased by at least 1 point: 152 patients (24.8%) without and 15 patients (31.2%) with severe nonadherence. In the univariate analyses, age, Black race, education, cigarette smoking, BMI, immunosuppressive treatment, SLEDAI‐2K, and nonquantifiable serum levels of HCQ were associated with risk of damage (Table 3). There was no statistically significant trend toward a higher risk of damage with severe nonadherence (hazard ratio [HR] 1.30; 95% CI 0.74–2.29; Figure 2). In the multivariate analyses, nonquantifiable serum levels of HCQ were independently associated with risk of damage (adjusted HR [aHR] 1.93, 95% CI 1.04–3.59, Table 3, Figure 2), along with age (aHR 1.02 per 1 year; 95% CI 1.01–1.03) and lower educational level (HR 1.94; 95% CI 1.43–2.64 for high school or less education, compared with postsecondary education) (data not shown).

**Table 3:** Hazard ratios (95% confidence intervals) for the risk of damage (≥1‐point increase of the SLICC damage index) in the five years after measurement of serum hydroxychloroquine level at time zero (T0) (n = 660)*

Demographic data and comorbidities	Overall, n = 660	≥1‐point increase in SLICC damage index within 5 years		
		n events (%) or mean ± SD, n = 167	Univariate OR (95% CI)	Multivariate OR (95% CI)
Age at serum sample, mean ± SD, y	36.2 ± 13.5	39.0 ± 15.3	1.02 (1.01–1.03)	1.02 (1.01–1.03)
Male, n (%)	80 (12.7)	20 ± 12.0	1.04 (0.65–1.65)	
Black race, n (%)	107 (16.2)	36 ± 21.6	1.59 (1.10–2.30)	1.64 (1.11–2.42)
Education level, n (%)				
Post‐secondary	413 (62.6)	80 ± 47.9	Reference	Reference
High school or less	247 (37.4)	87 ± 52.1	2.02 (1.49–2.73)	1.92 (1.40–2.63)
Cigarette smoking, n (%)				
Non‐smoker	437 (66.2)	97 ± 58.1	Reference	
Current or past smoker	222 (33.6)	69 ± 41.3	1.52 (1.12–2.07)	
BMI, kg/m^2^, mean ± SD	25.8 ± 6.1	26.8 ± 6.5	1.03 (1.01–1.06)	
SLEDAI‐2K at T0, mean ± SD	4.8 ± 4.9	5.4 ± 5.5	1.03 (1.00–1.06)	1.03 (1.00–1.06)
Treatment at T0, n (%)				
Corticosteroids	438 (66.4)	118 ± 70.7	1.28 (0.92–1.79)	
Azathioprine	91 (13.8)	36 ± 21.6	2.01 (1.39–2.90)	1.85 (1.26–2.73)
Hydroxychloroquine				
Severe HCQ nonadherence	48 (7.3)	15 ± 9.0	1.47 (0.86–2.49)	1.30 (0.74–2.29)
Nonquantifiable serum levels	28 (4.2)	11 ± 6.6	1.99 (1.08–3.66)	

* Results are expressed as n (%) for categorical variables and mean ± SD for continuous variables. The following variables were included in the multivariate model: age, Black race, education level (post‐secondary; ≤ high school), SLEDAI‐2000, azathioprine, and severe HCQ nonadherence.

BMI, body mass index; CI, 95% confidence interval; HCQ, hydroxychloroquine; kg/m^2^, kilograms per square meter; OR, odds ratio; SD, standard deviation; SLICC, Systemic Lupus International Collaborating Clinics; SLEDAI‐2K, Systemic Lupus Erythematosus Disease Activity Index 2000.

We observed that the trajectories of damage accrual diverged at one, two, and three years and then tended to converge by five years (Figure 2), although precision was limited at that point. The risk of worsening damage was higher during the first year (aHR 4.26; 95% CI 1.40–13), between T0 and year 2 (aHR 3.54; 95% CI 1.83–6.86), and between T0 and year 3 (aHR 1.92; 95% CI 1.05–3.50). We also explored whether effects on damage differed according to whether it was likely related to uncontrolled disease activity or to treatment side effects.

Five years after T0, among patients with an SDI increase ≥1 point (n = 167), patients with severe nonadherence had treatment‐related damage (13.3% vs 33.6%) less frequently and disease‐related damage (100% vs 80.9%) more frequently, although these differences were not statistically significant (Supplementary Table 6).

### Severe nonadherence to HCQ at T0 and mortality at five years

In the five years after T0, 11 patients died, including the 3 of 48 patients with severe nonadherence. In the univariate analyses, the HR for the risk of death during this five‐year period was 5.41 (95% CI 1.43–20.39) for patients with severe nonadherence (Table 4). The reported causes of death for the three severely nonadherent patients were multiorgan failure due to SLE and cardiac tamponade, probable septic shock with end‐stage renal disease, and cardiorespiratory arrest with respiratory failure; the adherent patients died from cardiorespiratory failure (n = 3), sepsis (n = 2), pulmonary vasculitis (n = 1), or from unknown causes (n = 2).

**Table 4:** Hazard ratios (95% confidence intervals) for the risk of death in the five years after measurement of the serum hydroxychloroquine level at time zero (T0) (N = 660)*

Demographic data and comorbidities	Overall, n = 660	Death within 5 years	
		n events (%) or mean ± SD, n = 11	Univariate OR (95% CI)
Age at serum sample, years, mean ± SD	36.2 ± 13.5	44.8 ± 19.3	1.05 (1.01–1.09)
Male, n (%)	80 (12.7)	0 (0.0)	–
Black race, n (%)	107 (16.2)	2 (18.2)	1.31 (0.28–6.06)
Education level, n (%)			
Post‐secondary	413 (62.6)	5 (45.5)	Reference
High school or less	247 (37.4)	6 (54.5)	2.25 (0.69–7.37)
Cigarette smoking, n (%)			
Non‐smoker	437 (66.2)	5 (45.5)	Reference
Current or past smoker	222 (33.6)	6 (54.5)	2.63 (0.80–8.61)
BMI, kg/m^2^, mean ± SD	25.79 ± 6.1	26.3 ± 6.8	1.03 (0.93–1.14)
SLEDAI‐2K at T0, mean ± SD	4.8 ± 4.9	6.6 ± 6.1	1.07 (0.97–1.18)
Corticosteroids	438 ± 66.4	11 ± 100.0	1.42 (0.99–2.06)
Azathioprine	91 ± 13.8	2 ± 18.2	1.66 (0.36–7.69)
Hydroxychloroquine			
Severe HCQ nonadherence	48 ± 7.3	3 ± 27.3	5.41 (1.43–20.39)
Nonquantifiable serum levels	28 ± 4.2	1 ± 9.1	2.87 (0.37–22.38)

* Results are expressed as n (%) for categorical variables and mean ± SD for continuous variables. BMI, body mass index; CI, 95% confidence interval; HCQ, hydroxychloroquine; HR, hazard ratio; kg/m^2^, kilograms per square meter; OR, odds ratio; SD, standard deviation; SLEDAI‐2000, Systemic Lupus Erythematosus Disease Activity Index 2000.

## DISCUSSION

Our study showed a 7.3% rate of severe nonadherence based on HCQ levels at enrollment in an inception cohort of patients with lupus. In this large, international, multicenter, longitudinal cohort, severe nonadherence to HCQ was independently associated with the risk of an SLE flare in the following year, with damage at one, two, and three years and with five‐year mortality. Nonquantifiable serum HCQ levels were also associated with the risk of damage within five years.

Our results are concordant with the 7% nonadherence rate reported in a previous French series^16^ and are lower than other similar published studies of HCQ blood or serum levels,^27^, ^28^, ^45^ which found nonadherence rates as high as 29%. This may be explained by differences (in age distribution, SLE duration, etc) in study populations, because adherence is known to vary by age and decrease over time.^46^ Different threshold HCQ concentrations defining nonadherence might also contribute to these discrepancies. Other studies, mainly based on self‐administered questionnaires, found higher nonadherence rates, but questionnaires probably measure different nonadherence patterns (tablets missed relatively infrequently), as reflected by the moderate correlation between these methods.^30^

The only characteristic associated with severe nonadherence was azathioprine use at T0, with nonsignificant trends toward higher SLEDAI‐2K, higher BMI, and higher proportions of female and Black patients among those nonadherent. The fact that azathioprine was prescribed before or concomitantly with HCQ for most nonadherent patients makes the likelihood of azathioprine prescription as a consequence of nonadherence very unlikely. A previous French series found a higher SLEDAI‐2K score was the main factor that differentiated adherent from nonadherent patients.^16^ An international longitudinal study found that younger age, absence of steroid treatment, higher BMI, and unemployment independently predicted nonadherence defined by blood drug measurements.^30^ Some studies assessing nonadherence with self‐reported questionnaires have reported race/ethnicity, disease duration, low education, and/or younger age to be associated with nonadherence,^20^, ^21^, ^44^ but as stated above, self‐reported nonadherence might represent a different pattern, distinguishable from severe nonadherence defined by blood/serum levels. The absence of any predictive marker of severe nonadherence was unsurprising, given the poor correlation between nonadherence by drug level and by physician assessment that some of our group demonstrated in an international study.^30^ This suggests the importance of HCQ measurements for unmasking severe nonadherence.

HCQ has long been known to decrease SLE activity and prevent flares.^48^ Recently, a study from the SLICC group demonstrated higher SLE flare risk after HCQ discontinuation or taper versus maintenance.^49^ However, few studies have assessed the risk of SLE flares associated with HCQ nonadherence, although low blood and serum levels of HCQ are associated with increased SLE activity or subsequent systemic and renal flares during follow‐up.^16^, ^17^, ^25^, ^26^, ^33^, ^36^, ^51^, ^52^, ^53^ We found severe nonadherence was clearly associated with flare risk, defined by a SLEDAI–2K increase of 4 points or more, a new prednisone or other immunosuppressive prescription, or new renal involvement. The remarkable stability of the associations with each component of our composite outcome examined separately strengthens our findings.

The risk of damage (SDI increase) within five years did not differ significantly according to severe nonadherence, but the significance of the association at one, two, and three years suggests an association with early damage. Strikingly, two different kinetics of damage accrual (Figure 2) were observed: early damage in severely nonadherent patients and the convergence of these curves due to later damage accrual in adherent patients. Damage captured by SDI can be related to the disease itself but also to its treatment; corticosteroid use is associated with the transition from no damage to damage and with greater pre‐existing damage.^8^ Treatment‐related damage includes diabetes, muscle atrophy, osteoporosis, avascular necrosis, or cataract (possibly due to glucocorticoids), retinal damage (potentially due to HCQ), or premature infertility (potentially due to other immunosuppressive drugs). Such damage usually takes years to occur, whereas damage directly linked to SLE may occur earlier. To explain our findings, particularly the different curves of Figure 2, we hypothesized that treatment‐related damage occurred mainly in adherent patients and appeared after a few years, whereas other damage (often due to SLE activity) occurred more frequently and earlier in the disease in severely nonadherent patients. We indeed found such a tendency, albeit not statistically significant, possibly due to inadequate power. As our group and others have shown that damage in SLE predicts future damage accrual and mortality and that severe nonadherence appears to be a major and potentially modifiable risk factor for damage accrual, these findings strongly argue that severe nonadherence should be actively sought by assessing HCQ levels as a first step toward improving adherence.^17^, ^53^, ^54^

Finally, severe nonadherence to HCQ was associated with the risk of death, even though the small number of events prevented any multivariate analyses. Although HCQ's role in reducing mortality in patients with SLE is known,^11^ to our knowledge, this is the first time that a link between severe nonadherence and mortality has been demonstrated in patients with SLE. Admittedly, the inability to use multivariate models limits the strength of our conclusion.

Some limitations must be acknowledged: First, including only patients prescribed HCQ for at least three months at inclusion and with an available sample excluded more than 60% of the cohort (including 40% initially included less than three months after their SLE diagnosis). Nonetheless, our study population differed only slightly from the excluded population (see Supplemental Materials).

Second, to define severe nonadherence, we used prespecified thresholds, based on the prescribed HCQ daily dosage, as our rate of patients taking 200 mg/day was higher than in previous studies. Thus, if the daily dose was not 200 or 400 mg, we had to round to the nearest category, which could have introduced some bias. However, among the patients with 300 mg/day (n = 62), fewer than 5% were considered nonadherent, below the 7.3% rate for the overall population. This finding suggests that this rounding did not artificially increase the number of nonadherent patients. Furthermore, choosing each of the two thresholds we used to define nonadherence regardless of HCQ dose led to very similar results, thus confirming the robustness of our findings.

Third, the validation of the bioanalytical method for HCQ assessment in serum samples according to the European Medicines Agency recommendations ensures the measurement's robustness.^55^ The literature include no data about the stability of HCQ in serum for a prolonged interval between sampling and assay. Nonetheless, the median serum HCQ levels we found were very close to reports from other studies and suggests that any potential degradation of HCQ would have had a limited impact on our results.

Fourth, seven patients were pregnant at T0. Although pregnancy can impact adherence and dosing, only one patient was considered severely nonadherent. The patient's undetectable serum level makes it unlikely that the conclusion of nonadherence is wrong. Furthermore, no pregnant patient had a flare or an increase in SDI during follow‐up. It is therefore unlikely that these pregnancies affected our results.

Fifth, we defined SLE flares by a composite endpoint: increased SLEDAI‐2K, new renal involvement, or a new prescription for prednisone and/or other immunosuppressive agent. Follow‐up visits and SLE activity assessment occurred yearly. Using only an increase in SLEDAI‐2K might not have captured an SLE flare occurring between two follow‐up visits, whereas new treatment or new renal involvement since the last visit might well reflect such a flare. Moreover, a similar composite endpoint has previously been used in SLICC cohort studies,^49^ and our findings remained significant when the SLEDAI‐2K increase and new treatments were assessed separately.

We acknowledge that we only had one serum HCQ measurement at T0—not repeated measurements. Thus, we could not consider patients with severe nonadherence to HCQ at T0 who became adherent during follow‐up visits or those in the inverse situation. To limit this bias, SLE flares were assessed in the year after T0. We assessed the risk of damage and mortality at five years and showed that severe nonadherence shown by a single measurement was associated with these risks, thus strengthening the demonstration of this measurement's utility, even when performed only once. Finally, two‐thirds of the patients took concomitant steroids, and one‐third had other concomitant immunosuppressive treatments. It is likely that at least some patients with severe nonadherence to HCQ were also nonadherent to other treatments, as previously shown.^16^ Unfortunately, it was not possible to measure adherence to other treatments; although the levels of some can be measured in serum samples, they reflect only very recent drug intake, in contrast to HCQ (and azathioprine metabolites), which have a long half‐life. In any case, regardless of adherence to the other drugs, HCQ nonadherence is easy to assess with blood or serum HCQ measurement and may well reflect global treatment adherence. Thus, interventions on HCQ nonadherence might also apply to other drugs if relevant.

Our study has several strengths, including the large cohort size and the multicenter design. Data were collected longitudinally, and very few were missing. The high number of events (disease flares or damage) provided sufficient statistical power to show associations. We also measured HCQ serum levels centrally and demonstrated that serum bank samples can be used when whole blood is not available, as previously suggested.^35^, ^36^

In conclusion, we demonstrated that severe nonadherence to HCQ is associated with unfavorable outcomes among patients with SLE, including flares, SLE damage, and death. As severe nonadherence is often unknown by the physician and because no predictive clinical or biological factors have been identified, our results underline the benefits of systematically testing to detect severe nonadherence and identify the patients at risk. Once uncovered, dedicating more resources and more time to these patients, and implementing specific strategies for them may help prevent SLE flares and damage and thus improve their long‐term prognosis.

## AUTHOR CONTRIBUTIONS

All authors were involved in drafting the article or revising it critically for important intellectual content, and all authors approved the final version to be published. Pr. Costedoat‐Chalumeau had full access to all of the data in the study and takes responsibility for the integrity of the data and the accuracy of the data analysis.

### Study conception and design

Nguyen, Blanchet, Urowitz, Hanly, Gordon, Bae, Romero‐Diaz, Clarke, Bernatsky, Wallace, Isenberg, Rahman, Merrill, Fortin, Gladman, Bruce, Petri, Ginzler, Dooley, Ramsey‐Goldman, Manzi, Jönsen, Alarcón, van Vollenhoven, Aranow, Le Guern, Mackay, Ruiz‐Irastorza, Lim, Inanc, Kalunian, Jacobsen, Peschken, Kamen, Askanase, Buyon, Costedoat‐Chalumeau.

### Acquisition of data

Nguyen, Blanchet, Costedoat‐Chalumeau.

### Analysis and interpretation of data

Nguyen, Blanchet, Clarke, Bernatsky, Gladman, Aranow, Buyon, Costedoat‐Chalumeau.

## Supporting information


Disclosure Form

**Appendix S1:** Supporting Information
