# Supplementary material for: Association Between Severe Nonadherence to Hydroxychloroquine and Systemic Lupus Erythematosus Flares, Damage, and Mortality in 660 Patients From the SLICC Inception Cohort
Source: Arthritis Rheumatol. 2023 Nov 13;75(12):2195–206. doi: 10.1002/art.42645 (PMC10792124; doi:10.1002/art.42645)
Supplement: Supplementary file 1 — Disclosure Form [file ART-75-2195-s001.pdf]

ICMJE DISCLOSURE FORM

Date: February 14th 2023
Your Name: Yann NGUYEN
Manuscript Title : Association between severe non-adherence to hydroxychloroquine and SLE flares, damage, and mortality in 660 patients from the SLICC Inception Cohort
Manuscript number (if known): ar-22-1545.R1

In the interest of transparency, we ask you to disclose all relationships/activities/interests listed below that are related to the content of your manuscript. "Related" means any relation with for-profit or not-for-profit third parties whose interests may be affected by the content of the manuscript. Disclosure represents a commitment to transparency and does not necessarily indicate a bias. If you are in doubt about whether to list a relationship/activity/interest, it is preferable that you do so.

The following questions apply to the author's relationships/activities/interests as they relate to the current manuscript only.

The author's relationships/activities/interests should be defined broadly. For example, if your manuscript pertains to the epidemiology of hypertension, you should declare all relationships with manufacturers of antihypertensive medication, even if that medication is not mentioned in the manuscript.

In item #1 below, report all support for the work reported in this manuscript without time limit. For all other items, the time frame for disclosure is the past 36 months.

		**Name all entities with whom you have this relationship or indicate none (add rows as needed)**	**Specifications/Comments (e.g., if payments were made to you or to your institution)**
		**Time frame: Since the initial planning of the work**	
1	All support for the present manuscript (e.g., funding, provision of study materials, medical writing, article processing charges, etc.) **No time limit for this item.**	_X__None	
		**Time frame: past 36 months**	
2	Grants or contracts from any entity (if not indicated in item #1 above).	_X__None	
3	Royalties or licenses	_X__None	
4	Consulting fees	_X__None	

5	Payment or honoraria for lectures, presentations, speakers bureaus, manuscript writing or educational events	_X__None	
6	Payment for expert testimony	_X__None	
7	Support for attending meetings and/or travel	_X__None	
8	Patents planned, issued or pending	_X__None	
9	Participation on a Data Safety Monitoring Board or Advisory Board	_X__None	
10	Leadership or fiduciary role in other board, society, committee or advocacy group, paid or unpaid	_X__None	
11	Stock or stock options	_X__None	
12	Receipt of equipment, materials, drugs, medical writing, gifts or other services	_X__None	
13	Other financial or non-financial interests	_X__None	

Please place an "X" next to the following statement to indicate your agreement:

_X__ I certify that I have answered every question and have not altered the wording of any of the questions on this form.

ICMJE DISCLOSURE FORM

Date: February 14th 2023
Your Name: Dr Benoit Blanchet
Manuscript Title : Association between severe non-adherence to hydroxychloroquine and SLE flares, damage, and mortality in 660 patients from the SLICC Inception Cohort
Manuscript number (if known): ar-22-1545.R1

In the interest of transparency, we ask you to disclose all relationships/activities/interests listed below that are related to the content of your manuscript. "Related" means any relation with for-profit or not-for-profit third parties whose interests may be affected by the content of the manuscript. Disclosure represents a commitment to transparency and does not necessarily indicate a bias. If you are in doubt about whether to list a relationship/activity/interest, it is preferable that you do so.

The following questions apply to the author's relationships/activities/interests as they relate to the current manuscript only.

The author's relationships/activities/interests should be defined broadly. For example, if your manuscript pertains to the epidemiology of hypertension, you should declare all relationships with manufacturers of antihypertensive medication, even if that medication is not mentioned in the manuscript.

In item #1 below, report all support for the work reported in this manuscript without time limit. For all other items, the time frame for disclosure is the past 36 months.

		**Name all entities with whom you have this relationship or indicate none (add rows as needed)**	**Specifications/Comments (e.g., if payments were made to you or to your institution)**
		**Time frame: Since the initial planning of the work**	
1	All support for the present manuscript (e.g., funding, provision of study materials, medical writing, article processing charges, etc.) **No time limit for this item.**	____None	
		**Time frame: past 36 months**	
2	Grants or contracts from any entity (if not indicated in item #1 above).	____None	
3	Royalties or licenses	____None	
4	Consulting fees	____None	

5	Payment or honoraria for lectures, presentations, speakers bureaus, manuscript writing or educational events	____None	
6	Payment for expert testimony	____None	
7	Support for attending meetings and/or travel	____None	
8	Patents planned, issued or pending	____None	
9	Participation on a Data Safety Monitoring Board or Advisory Board	____None	
10	Leadership or fiduciary role in other board, society, committee or advocacy group, paid or unpaid	____None	
11	Stock or stock options	____None	
12	Receipt of equipment, materials, drugs, medical writing, gifts or other services	____None	
13	Other financial or non-financial interests	____None	

Please place an "X" next to the following statement to indicate your agreement:

___ I certify that I have answered every question and have not altered the wording of any of the questions on this form. X

ICMJE DISCLOSURE FORM

Date: February 14th 2023
Your Name:___Murray Urowitz
Manuscript Title : Association between severe non-adherence to hydroxychloroquine and SLE flares, damage, and mortality in 660 patients from the SLICC Inception Cohort
Manuscript number (if known): ar-22-1545.R1

In the interest of transparency, we ask you to disclose all relationships/activities/interests listed below that are related to the content of your manuscript. "Related" means any relation with for-profit or not-for-profit third parties whose interests may be affected by the content of the manuscript. Disclosure represents a commitment to transparency and does not necessarily indicate a bias. If you are in doubt about whether to list a relationship/activity/interest, it is preferable that you do so.

The following questions apply to the author's relationships/activities/interests as they relate to the current manuscript only.

The author's relationships/activities/interests should be defined broadly. For example, if your manuscript pertains to the epidemiology of hypertension, you should declare all relationships with manufacturers of antihypertensive medication, even if that medication is not mentioned in the manuscript.

In item #1 below, report all support for the work reported in this manuscript without time limit. For all other items, the time frame for disclosure is the past 36 months.

		**Name all entities with whom you have this relationship or indicate none (add rows as needed)**	**Specifications/Comments (e.g., if payments were made to you or to your institution)**
**Time frame: Since the initial planning of the work**			
1	All support for the present manuscript (e.g., funding, provision of study materials, medical writing, article processing charges, etc.) **No time limit for this item.**	_X__None	
**Time frame: past 36 months**			
2	Grants or contracts from any entity (if not indicated in item #1 above).	_X__None	
3	Royalties or licenses	_X__None	
4	Consulting fees	_X__None	

5	Payment or honoraria for lectures, presentations, speakers bureaus, manuscript writing or educational events	_X__None	
6	Payment for expert testimony	_X__None	
7	Support for attending meetings and/or travel	_X__None	
8	Patents planned, issued or pending	_X__None	
9	Participation on a Data Safety Monitoring Board or Advisory Board	_X__None	
10	Leadership or fiduciary role in other board, society, committee or advocacy group, paid or unpaid	_X__None	
11	Stock or stock options	_X__None	
12	Receipt of equipment, materials, drugs, medical writing, gifts or other services	_X__None	
13	Other financial or non-financial interests	_X__None	

Please place an "X" next to the following statement to indicate your agreement:

_X__ I certify that I have answered every question and have not altered the wording of any of the questions on this form.

ICMJE DISCLOSURE FORM

Date: February 14th 2023
Your Name:____John Hanly______________________
Manuscript Title : Association between severe non-adherence to hydroxychloroquine and SLE flares, damage, and mortality in 660 patients from the SLICC Inception Cohort
Manuscript number (if known): ar-22-1545.R1

In the interest of transparency, we ask you to disclose all relationships/activities/interests listed below that are related to the content of your manuscript. "Related" means any relation with for-profit or not-for-profit third parties whose interests may be affected by the content of the manuscript. Disclosure represents a commitment to transparency and does not necessarily indicate a bias. If you are in doubt about whether to list a relationship/activity/interest, it is preferable that you do so.

The following questions apply to the author's relationships/activities/interests as they relate to the current manuscript only.

The author's relationships/activities/interests should be defined broadly. For example, if your manuscript pertains to the epidemiology of hypertension, you should declare all relationships with manufacturers of antihypertensive medication, even if that medication is not mentioned in the manuscript.

In item #1 below, report all support for the work reported in this manuscript without time limit. For all other items, the time frame for disclosure is the past 36 months.

		**Name all entities with whom you have this relationship or indicate none (add rows as needed)**	**Specifications/Comments (e.g., if payments were made to you or to your institution)**
		**Time frame: Since the initial planning of the work**	
1	All support for the present manuscript (e.g., funding, provision of study materials, medical writing, article processing charges, etc.) **No time limit for this item.**	_X__None	
		**Time frame: past 36 months**	
2	Grants or contracts from any entity (if not indicated in item #1 above).	_X__None	
3	Royalties or licenses	_X__None	
4	Consulting fees	_X__None	

5	Payment or honoraria for lectures, presentations, speakers bureaus, manuscript writing or educational events	_X__None	
6	Payment for expert testimony	_X__None	
7	Support for attending meetings and/or travel	_X__None	
8	Patents planned, issued or pending	_X__None	
9	Participation on a Data Safety Monitoring Board or Advisory Board	_X__None	
10	Leadership or fiduciary role in other board, society, committee or advocacy group, paid or unpaid	_X__None	
11	Stock or stock options	_X__None	
12	Receipt of equipment, materials, drugs, medical writing, gifts or other services	_X__None	
13	Other financial or non-financial interests	_X__None	

Please place an "X" next to the following statement to indicate your agreement:

_X_ I certify that I have answered every question and have not altered the wording of any of the questions on this form.

14 February 2023

ICMJE DISCLOSURE FORM

Date: February 14th 2023
Your Name:_Caroline Gordon
Manuscript Title : Association between severe non-adherence to hydroxychloroquine and SLE flares, damage, and mortality in 660 patients from the SLICC Inception Cohort
Manuscript number (if known): ar-22-1545.R1

In the interest of transparency, we ask you to disclose all relationships/activities/interests listed below that are related to the content of your manuscript. "Related" means any relation with for-profit or not-for-profit third parties whose interests may be affected by the content of the manuscript. Disclosure represents a commitment to transparency and does not necessarily indicate a bias. If you are in doubt about whether to list a relationship/activity/interest, it is preferable that you do so.

The following questions apply to the author's relationships/activities/interests as they relate to the current manuscript only.

The author's relationships/activities/interests should be defined broadly. For example, if your manuscript pertains to the epidemiology of hypertension, you should declare all relationships with manufacturers of antihypertensive medication, even if that medication is not mentioned in the manuscript.

In item #1 below, report all support for the work reported in this manuscript without time limit. For all other items, the time frame for disclosure is the past 36 months.

		**Name all entities with whom you have this relationship or indicate none (add rows as needed)**	**Specifications/Comments (e.g., if payments were made to you or to your institution)**
**Time frame: Since the initial planning of the work**			
1	All support for the present manuscript (e.g., funding, provision of study materials, medical writing, article processing charges, etc.) **No time limit for this item.**	____Lupus UK	Payments to University of Birmingham to support nurse coordinator
**Time frame: past 36 months**			
2	Grants or contracts from any entity (if not indicated in item #1 above).	_X__None	
3	Royalties or licenses	_X__None	
4	Consulting fees	Alumis	Consultancy fees to self

		Amgen	Consultancy fees to self
		Astra-Zeneca<br>Sanofi<br>UCB	Consultancy fees to self<br>Consultancy fees to self<br>Consultancy fees to self
5	Payment or honoraria for lectures, presentations, speakers bureaus, manuscript writing or educational events	_X__None	
6	Payment for expert testimony	_X__None	
7	Support for attending meetings and/or travel	_X__None	
8	Patents planned, issued or pending	_X__None	
9	Participation on a Data Safety Monitoring Board or Advisory Board	_X__None	
10	Leadership or fiduciary role in other board, society, committee or advocacy group, paid or unpaid	_X__None	
11	Stock or stock options	_X__None	
12	Receipt of equipment, materials, drugs, medical writing, gifts or other services	_X__None	
13	Other financial or non-financial interests	_X__None	

Please place an "X" next to the following statement to indicate your agreement:

_X__ I certify that I have answered every question and have not altered the wording of any of the questions on this form.

ICMJE DISCLOSURE FORM

Date: February 14th 2023

Your Name:_Sang-Cheol Bae_____________________

Manuscript Title : Association between severe non-adherence to hydroxychloroquine and SLE flares, damage, and mortality in 660 patients from the SLICC Inception Cohort

Manuscript number (if known): ar-22-1545.R1

In the interest of transparency, we ask you to disclose all relationships/activities/interests listed below that are related to the content of your manuscript. "Related" means any relation with for-profit or not-for-profit third parties whose interests may be affected by the content of the manuscript. Disclosure represents a commitment to transparency and does not necessarily indicate a bias. If you are in doubt about whether to list a relationship/activity/interest, it is preferable that you do so.

The following questions apply to the author's relationships/activities/interests as they relate to the current manuscript only.

The author's relationships/activities/interests should be defined broadly. For example, if your manuscript pertains to the epidemiology of hypertension, you should declare all relationships with manufacturers of antihypertensive medication, even if that medication is not mentioned in the manuscript.

In item #1 below, report all support for the work reported in this manuscript without time limit. For all other items, the time frame for disclosure is the past 36 months.

		**Name all entities with whom you have this relationship or indicate none (add rows as needed)**	**Specifications/Comments (e.g., if payments were made to you or to your institution)**
		**Time frame: Since the initial planning of the work**	
1	All support for the present manuscript (e.g., funding, provision of study materials, medical writing, article processing charges, etc.) **No time limit for this item.**	_X__None	
		**Time frame: past 36 months**	
2	Grants or contracts from any entity (if not indicated in item #1 above).	_X__None	
3	Royalties or licenses	_X__None	
4	Consulting fees	_X__None	

5	Payment or honoraria for lectures, presentations, speakers bureaus, manuscript writing or educational events	_X__None	
6	Payment for expert testimony	_X__None	
7	Support for attending meetings and/or travel	_X__None	
8	Patents planned, issued or pending	_X__None	
9	Participation on a Data Safety Monitoring Board or Advisory Board	_X__None	
10	Leadership or fiduciary role in other board, society, committee or advocacy group, paid or unpaid	_X__None	
11	Stock or stock options	_X__None	
12	Receipt of equipment, materials, drugs, medical writing, gifts or other services	_X__None	
13	Other financial or non-financial interests	_X__None	

Please place an "X" next to the following statement to indicate your agreement:

_X_ I certify that I have answered every question and have not altered the wording of any of the questions on this form.

ICMJE DISCLOSURE FORM

Date: February 14th 2023
Your Name: JUANITA ROMERO-DÍAZ
Manuscript Title : Association between severe non-adherence to hydroxychloroquine and SLE flares, damage, and mortality in 660 patients from the SLICC Inception Cohort
Manuscript number (if known): ar-22-1545.R1

In the interest of transparency, we ask you to disclose all relationships/activities/interests listed below that are related to the content of your manuscript. "Related" means any relation with for-profit or not-for-profit third parties whose interests may be affected by the content of the manuscript. Disclosure represents a commitment to transparency and does not necessarily indicate a bias. If you are in doubt about whether to list a relationship/activity/interest, it is preferable that you do so.

The following questions apply to the author's relationships/activities/interests as they relate to the current manuscript only.

The author's relationships/activities/interests should be defined broadly. For example, if your manuscript pertains to the epidemiology of hypertension, you should declare all relationships with manufacturers of antihypertensive medication, even if that medication is not mentioned in the manuscript.

In item #1 below, report all support for the work reported in this manuscript without time limit. For all other items, the time frame for disclosure is the past 36 months.

		Name all entities with whom you have this relationship or indicate none (add rows as needed)	Specifications/Comments (e.g., if payments were made to you or to your institution)
		**Time frame: Since the initial planning of the work**	
1	All support for the present manuscript (e.g., funding, provision of study materials, medical writing, article processing charges, etc.) **No time limit for this item.**	✓ None	
		**Time frame: past 36 months**	
2	Grants or contracts from any entity (if not indicated in item #1 above).	✓ None	
3	Royalties or licenses	✓ None	
4	Consulting fees	✓ None	

5	Payment or honoraria for lectures, presentations, speakers bureaus, manuscript writing or educational events	✓ None	
6	Payment for expert testimony	✓ None	
7	Support for attending meetings and/or travel	✓ None	
8	Patents planned, issued or pending	✓ None	
9	Participation on a Data Safety Monitoring Board or Advisory Board	✓ None	
10	Leadership or fiduciary role in other board, society, committee or advocacy group, paid or unpaid	✓ None	
11	Stock or stock options	✓ None	
12	Receipt of equipment, materials, drugs, medical writing, gifts or other services	✓ None	
13	Other financial or non-financial interests	✓ None	

Please place an "X" next to the following statement to indicate your agreement:

X **I certify that I have answered every question and have not altered the wording of any of the questions on this form.**

ICMJE DISCLOSURE FORM

Date: February 14th 2023
Your Name: Jorge Sánchez-Guerrero
Manuscript Title: Association between severe non-adherence to hydroxychloroquine and SLE flares, damage, and mortality in 660 patients from the SLICC Inception Cohort
Manuscript number (if known): ar-22-1545.R1

In the interest of transparency, we ask you to disclose all relationships/activities/interests listed below that are related to the content of your manuscript. "Related" means any relation with for-profit or not-for-profit third parties whose interests may be affected by the content of the manuscript. Disclosure represents a commitment to transparency and does not necessarily indicate a bias. If you are in doubt about whether to list a relationship/activity/interest, it is preferable that you do so.

The following questions apply to the author's relationships/activities/interests as they relate to the current manuscript only.

The author's relationships/activities/interests should be defined broadly. For example, if your manuscript pertains to the epidemiology of hypertension, you should declare all relationships with manufacturers of antihypertensive medication, even if that medication is not mentioned in the manuscript.

In item #1 below, report all support for the work reported in this manuscript without time limit. For all other items, the time frame for disclosure is the past 36 months.

		**Name all entities with whom you have this relationship or indicate none (add rows as needed)**	**Specifications/Comments (e.g., if payments were made to you or to your institution)**
		**Time frame: Since the initial planning of the work**	
1	All support for the present manuscript (e.g., funding, provision of study materials, medical writing, article processing charges, etc.) **No time limit for this item.**	____None	
		**Time frame: past 36 months**	
2	Grants or contracts from any entity (if not indicated in item #1 above).	____None	
3	Royalties or licenses	____None	
4	Consulting fees	____None	

5	Payment or honoraria for lectures, presentations, speakers bureaus, manuscript writing or educational events	____None	
6	Payment for expert testimony	____None	
7	Support for attending meetings and/or travel	____None	
8	Patents planned, issued or pending	____None	
9	Participation on a Data Safety Monitoring Board or Advisory Board	____None	
10	Leadership or fiduciary role in other board, society, committee or advocacy group, paid or unpaid	____None	
11	Stock or stock options	____None	
12	Receipt of equipment, materials, drugs, medical writing, gifts or other services	____None	
13	Other financial or non-financial interests	____None	

Please place an "X" next to the following statement to indicate your agreement:

_✓_ **I certify that I have answered every question and have not altered the wording of any of the questions on this form.**

ICMJE DISCLOSURE FORM

Date: February 14th 2023
Your Name:___Ann Clarke_______________________
Manuscript Title : Association between severe non-adherence to hydroxychloroquine and SLE flares, damage, and mortality in 660 patients from the SLICC Inception Cohort
Manuscript number (if known): ar-22-1545.R1

In the interest of transparency, we ask you to disclose all relationships/activities/interests listed below that are related to the content of your manuscript. "Related" means any relation with for-profit or not-for-profit third parties whose interests may be affected by the content of the manuscript. Disclosure represents a commitment to transparency and does not necessarily indicate a bias. If you are in doubt about whether to list a relationship/activity/interest, it is preferable that you do so.

The following questions apply to the author's relationships/activities/interests as they relate to the current manuscript only.

The author's relationships/activities/interests should be defined broadly. For example, if your manuscript pertains to the epidemiology of hypertension, you should declare all relationships with manufacturers of antihypertensive medication, even if that medication is not mentioned in the manuscript.

In item #1 below, report all support for the work reported in this manuscript without time limit. For all other items, the time frame for disclosure is the past 36 months.

		**Name all entities with whom you have this relationship or indicate none (add rows as needed)**	**Specifications/Comments (e.g., if payments were made to you or to your institution)**
		**Time frame: Since the initial planning of the work**	
1	All support for the present manuscript (e.g., funding, provision of study materials, medical writing, article processing charges, etc.) **No time limit for this item.**	_X__None	
		**Time frame: past 36 months**	
2	Grants or contracts from any entity (if not indicated in item #1 above).	_X__None	
3	Royalties or licenses	_X__None	
4	Consulting fees	_X__None	

5	Payment or honoraria for lectures, presentations, speakers bureaus, manuscript writing or educational events	_X__None	
6	Payment for expert testimony	_X__None	
7	Support for attending meetings and/or travel	_X__None	
8	Patents planned, issued or pending	_X__None	
9	Participation on a Data Safety Monitoring Board or Advisory Board	_X__None	
10	Leadership or fiduciary role in other board, society, committee or advocacy group, paid or unpaid	_X__None	
11	Stock or stock options	_X__None	
12	Receipt of equipment, materials, drugs, medical writing, gifts or other services	_X__None	
13	Other financial or non-financial interests	_X__None	

Please place an "X" next to the following statement to indicate your agreement:

__X_ I certify that I have answered every question and have not altered the wording of any of the questions on this form.

ICMJE DISCLOSURE FORM

Date: February 14th 2023
Your Name: Sasha Bernatsky
Manuscript Title : Association between severe non-adherence to hydroxychloroquine and SLE flares, damage, and mortality in 660 patients from the SLICC Inception Cohort
Manuscript number (if known): ar-22-1545.R1

In the interest of transparency, we ask you to disclose all relationships/activities/interests listed below that are related to the content of your manuscript. "Related" means any relation with for-profit or not-for-profit third parties whose interests may be affected by the content of the manuscript. Disclosure represents a commitment to transparency and does not necessarily indicate a bias. If you are in doubt about whether to list a relationship/activity/interest, it is preferable that you do so.

The following questions apply to the author's relationships/activities/interests as they relate to the current manuscript only.

The author's relationships/activities/interests should be defined broadly. For example, if your manuscript pertains to the epidemiology of hypertension, you should declare all relationships with manufacturers of antihypertensive medication, even if that medication is not mentioned in the manuscript.

In item #1 below, report all support for the work reported in this manuscript without time limit. For all other items, the time frame for disclosure is the past 36 months.

		**Name all entities with whom you have this relationship or indicate none (add rows as needed)**	**Specifications/Comments (e.g., if payments were made to you or to your institution)**
		**Time frame: Since the initial planning of the work**	
1	All support for the present manuscript (e.g., funding, provision of study materials, medical writing, article processing charges, etc.) **No time limit for this item.**	_x___None	
		**Time frame: past 36 months**	
2	Grants or contracts from any entity (if not indicated in item #1 above).	__x__None	
3	Royalties or licenses	__x__None	
4	Consulting fees	__x__None	
5	Payment or honoraria for lectures, presentations,	_X__None	

	speakers bureaus, manuscript writing or educational events		
6	Payment for expert testimony	__x__None	
7	Support for attending meetings and/or travel	_x___None	
8	Patents planned, issued or pending	_x___None	
9	Participation on a Data Safety Monitoring Board or Advisory Board	_x___None	
10	Leadership or fiduciary role in other board, society, committee or advocacy group, paid or unpaid	__x__None	
11	Stock or stock options	__x__None	
12	Receipt of equipment, materials, drugs, medical writing, gifts or other services	__x__None	
13	Other financial or non-financial interests	_x___None	

Please place an "X" next to the following statement to indicate your agreement:

_x__ I certify that I have answered every question and have not altered the wording of any of the questions on this form.

ICMJE DISCLOSURE FORM

Date: February 14th 2023
Your Name:__Daniel J Wallace M.D.
Manuscript Title : Association between severe non-adherence to hydroxychloroquine and SLE flares, damage, and mortality in 660 patients from the SLICC Inception Cohort
Manuscript number (if known): ar-22-1545.R1

In the interest of transparency, we ask you to disclose all relationships/activities/interests listed below that are related to the content of your manuscript. "Related" means any relation with for-profit or not-for-profit third parties whose interests may be affected by the content of the manuscript. Disclosure represents a commitment to transparency and does not necessarily indicate a bias. If you are in doubt about whether to list a relationship/activity/interest, it is preferable that you do so.

The following questions apply to the author's relationships/activities/interests as they relate to the current manuscript only.

The author's relationships/activities/interests should be defined broadly. For example, if your manuscript pertains to the epidemiology of hypertension, you should declare all relationships with manufacturers of antihypertensive medication, even if that medication is not mentioned in the manuscript.

In item #1 below, report all support for the work reported in this manuscript without time limit. For all other items, the time frame for disclosure is the past 36 months.

		**Name all entities with whom you have this relationship or indicate none (add rows as needed)**	**Specifications/Comments (e.g., if payments were made to you or to your institution)**
**Time frame: Since the initial planning of the work**			
1	All support for the present manuscript (e.g., funding, provision of study materials, medical writing, article processing charges, etc.) **No time limit for this item.**	_X__None	
**Time frame: past 36 months**			
2	Grants or contracts from any entity (if not indicated in item #1 above).	_X__None	
3	Royalties or licenses	_X__None	
4	Consulting fees	_X__None	

5	Payment or honoraria for lectures, presentations, speakers bureaus, manuscript writing or educational events	_X__None	
6	Payment for expert testimony	_X__None	
7	Support for attending meetings and/or travel	_X__None	
8	Patents planned, issued or pending	_X__None	
9	Participation on a Data Safety Monitoring Board or Advisory Board	_X__None	
10	Leadership or fiduciary role in other board, society, committee or advocacy group, paid or unpaid	_X__None	
11	Stock or stock options	_X__None	
12	Receipt of equipment, materials, drugs, medical writing, gifts or other services	_X__None	
13	Other financial or non-financial interests	_X__None	

Please place an "X" next to the following statement to indicate your agreement:

_X__ I certify that I have answered every question and have not altered the wording of any of the questions on this form.

ICMJE DISCLOSURE FORM

Date: February 14th 2023
Your Name:__DAVID ISENBERG________________________
Manuscript Title : Association between severe non-adherence to hydroxychloroquine and SLE flares, damage, and mortality in 660 patients from the SLICC Inception Cohort
Manuscript number (if known): ar-22-1545.R1

In the interest of transparency, we ask you to disclose all relationships/activities/interests listed below that are related to the content of your manuscript. "Related" means any relation with for-profit or not-for-profit third parties whose interests may be affected by the content of the manuscript. Disclosure represents a commitment to transparency and does not necessarily indicate a bias. If you are in doubt about whether to list a relationship/activity/interest, it is preferable that you do so.

The following questions apply to the author's relationships/activities/interests as they relate to the current manuscript only.

The author's relationships/activities/interests should be defined broadly. For example, if your manuscript pertains to the epidemiology of hypertension, you should declare all relationships with manufacturers of antihypertensive medication, even if that medication is not mentioned in the manuscript.

In item #1 below, report all support for the work reported in this manuscript without time limit. For all other items, the time frame for disclosure is the past 36 months.

		**Name all entities with whom you have this relationship or indicate none (add rows as needed)**	**Specifications/Comments (e.g., if payments were made to you or to your institution)**
		**Time frame: Since the initial planning of the work**	
1	All support for the present manuscript (e.g., funding, provision of study materials, medical writing, article processing charges, etc.) **No time limit for this item.**	_X__None	
		**Time frame: past 36 months**	
2	Grants or contracts from any entity (if not indicated in item #1 above).	_X__None	
3	Royalties or licenses	_X__None	
4	Consulting fees	_X__None	

5	Payment or honoraria for lectures, presentations, speakers bureaus, manuscript writing or educational events	Astra Zeneca, Vera Therapeutics, Amgen, Merck Serono, Servier and UCB Pharma	'I have received honoraria from Astra Zeneca, Vera Therapeutics, Amgen, Merck Serono, Servier and UCB Pharma. These honoraria are passed onto a local arthritis charity'
6	Payment for expert testimony	_X__None	
7	Support for attending meetings and/or travel	_X__None	
8	Patents planned, issued or pending	_X__None	
9	Participation on a Data Safety Monitoring Board or Advisory Board	_X__None	
10	Leadership or fiduciary role in other board, society, committee or advocacy group, paid or unpaid	_X__None	
11	Stock or stock options	_X__None	
12	Receipt of equipment, materials, drugs, medical writing, gifts or other services	_X__None	
13	Other financial or non-financial interests	_X__None	

Please place an "X" next to the following statement to indicate your agreement:

___ I certify that I have answered every question and have not altered the wording of any of the questions on this form. X

ICMJE DISCLOSURE FORM

Date: February 14th 2023
Your Name:____ANISUR RAHMAN______________________
Manuscript Title : Association between severe non-adherence to hydroxychloroquine and SLE flares, damage, and mortality in 660 patients from the SLICC Inception Cohort
Manuscript number (if known): ar-22-1545.R1

In the interest of transparency, we ask you to disclose all relationships/activities/interests listed below that are related to the content of your manuscript. "Related" means any relation with for-profit or not-for-profit third parties whose interests may be affected by the content of the manuscript. Disclosure represents a commitment to transparency and does not necessarily indicate a bias. If you are in doubt about whether to list a relationship/activity/interest, it is preferable that you do so.

The following questions apply to the author's relationships/activities/interests as they relate to the current manuscript only.

The author's relationships/activities/interests should be defined broadly. For example, if your manuscript pertains to the epidemiology of hypertension, you should declare all relationships with manufacturers of antihypertensive medication, even if that medication is not mentioned in the manuscript.

In item #1 below, report all support for the work reported in this manuscript without time limit. For all other items, the time frame for disclosure is the past 36 months.

		**Name all entities with whom you have this relationship or indicate none (add rows as needed)**	**Specifications/Comments (e.g., if payments were made to you or to your institution)**
		**Time frame: Since the initial planning of the work**	
1	All support for the present manuscript (e.g., funding, provision of study materials, medical writing, article processing charges, etc.) **No time limit for this item.**	_X__None	
		**Time frame: past 36 months**	
2	Grants or contracts from any entity (if not indicated in item #1 above).	_X__None	
3	Royalties or licenses	_X__None	
4	Consulting fees	_X__None	

5	Payment or honoraria for lectures, presentations, speakers bureaus, manuscript writing or educational events	_X__None	
6	Payment for expert testimony	_X__None	
7	Support for attending meetings and/or travel	_X__None	
8	Patents planned, issued or pending	_X__None	
9	Participation on a Data Safety Monitoring Board or Advisory Board	_X__None	
10	Leadership or fiduciary role in other board, society, committee or advocacy group, paid or unpaid	_X__None	
11	Stock or stock options	_X__None	
12	Receipt of equipment, materials, drugs, medical writing, gifts or other services	_X__None	
13	Other financial or non-financial interests	_X__None	

Please place an "X" next to the following statement to indicate your agreement:

_X__ I certify that I have answered every question and have not altered the wording of any of the questions on this form.

ICMJE DISCLOSURE FORM

Date: February 14th 2023
Your Name:____Joann Merrill______________________
Manuscript Title : Association between severe non-adherence to hydroxychloroquine and SLE flares, damage, and mortality in 660 patients from the SLICC Inception Cohort
Manuscript number (if known): ar-22-1545.R1

In the interest of transparency, we ask you to disclose all relationships/activities/interests listed below that are related to the content of your manuscript. "Related" means any relation with for-profit or not-for-profit third parties whose interests may be affected by the content of the manuscript. Disclosure represents a commitment to transparency and does not necessarily indicate a bias. If you are in doubt about whether to list a relationship/activity/interest, it is preferable that you do so.

The following questions apply to the author's relationships/activities/interests as they relate to the current manuscript only.

The author's relationships/activities/interests should be defined broadly. For example, if your manuscript pertains to the epidemiology of hypertension, you should declare all relationships with manufacturers of antihypertensive medication, even if that medication is not mentioned in the manuscript.

In item #1 below, report all support for the work reported in this manuscript without time limit. For all other items, the time frame for disclosure is the past 36 months.

		**Name all entities with whom you have this relationship or indicate none (add rows as needed)**	**Specifications/Comments (e.g., if payments were made to you or to your institution)**
		**Time frame: Since the initial planning of the work**	
1	All support for the present manuscript (e.g., funding, provision of study materials, medical writing, article processing charges, etc.) **No time limit for this item.**	_X__None	
		**Time frame: past 36 months**	
2	Grants or contracts from any entity (if not indicated in item #1 above).	_X__None	
3	Royalties or licenses	_X__None	
4	Consulting fees	_X__None	

5	Payment or honoraria for lectures, presentations, speakers bureaus, manuscript writing or educational events	_X__None	
6	Payment for expert testimony	_X__None	
7	Support for attending meetings and/or travel	_X__None	
8	Patents planned, issued or pending	_X__None	
9	Participation on a Data Safety Monitoring Board or Advisory Board	_X__None	
10	Leadership or fiduciary role in other board, society, committee or advocacy group, paid or unpaid	_X__None	
11	Stock or stock options	_X__None	
12	Receipt of equipment, materials, drugs, medical writing, gifts or other services	_X__None	
13	Other financial or non-financial interests	_X__None	

Please place an "X" next to the following statement to indicate your agreement:

_X__ I certify that I have answered every question and have not altered the wording of any of the questions on this form.

ICMJE DISCLOSURE FORM

Date: February 14th 2023
Your Name:___**Paul R Fortin**______________________
Manuscript Title : Association between severe non-adherence to hydroxychloroquine and SLE flares, damage, and mortality in 660 patients from the SLICC Inception Cohort
Manuscript number (if known): ar-22-1545.R1

In the interest of transparency, we ask you to disclose all relationships/activities/interests listed below that are related to the content of your manuscript. "Related" means any relation with for-profit or not-for-profit third parties whose interests may be affected by the content of the manuscript. Disclosure represents a commitment to transparency and does not necessarily indicate a bias. If you are in doubt about whether to list a relationship/activity/interest, it is preferable that you do so.

The following questions apply to the author's relationships/activities/interests as they relate to the current manuscript only.

The author's relationships/activities/interests should be defined broadly. For example, if your manuscript pertains to the epidemiology of hypertension, you should declare all relationships with manufacturers of antihypertensive medication, even if that medication is not mentioned in the manuscript.

In item #1 below, report all support for the work reported in this manuscript without time limit. For all other items, the time frame for disclosure is the past 36 months.

		Name all entities with whom you have this relationship or indicate none (add rows as needed)	Specifications/Comments (e.g., if payments were made to you or to your institution)
		Time frame: Since the initial planning of the work	
1	All support for the present manuscript (e.g., funding, provision of study materials, medical writing, article processing charges, etc.) **No time limit for this item.**	__X__None	
		Time frame: past 36 months	
2	Grants or contracts from any entity (if not indicated in item #1 above).	____None	1) Collaborator. The implication of PIGF in cutaneous fibrosis. Canadian Institutes of Health Research (CIHR).PI: Véronique Moulin. Co-Investigator
			2) Co-Investigator. FlexCare: Co-developing a Flexible Care Delivery Model for Inflammatory Arthritis. The Arthritis Society. 450 000 CAD. PI: Diane Lacaille. Co-Investigator: Barber C, Barnabe C, Li Lc, Bansback N,

			Bartlett S, Da Costa D, Hoens A, Fortin P, Michou L, Colmegna I, Hazlewood G, Koehn C.
			3) Co-Investigator. Megakaryocyte: a new player in systemic lupus erythematosus. Lupus Fondation of America, Inc. (USA) / 2022 Lupus Canada Catalyst Grant. 40,000 CAD PI: Éric Boilard<br>4) Co-Investigator. Personalized therapy in lupus pregnancies. Canadian Rheumatology association (CRA). 114,000 CAD PI: Ann Elaine Clarke. Co-investigator: Bernatsky S, Touma Z, Grunbaum A, Hanly J, Peschken C, Laskin C, Costedoat N, Urowitz M, Proulx L.<br>5) Co-Investigator. Impact of vascular injury derived exosomes in Lupus Nephritis. Canadian Institutes of Health Research (CIHR). 577,575 CAD PI: Mélanie Dieudé. Co-investigator: Éric Boilard, Héloise Cardinal<br>6) Principal Investigator. Booster dose of mRNA SARS-CoV-2 vaccine versus non mRNA vaccine for people living with systemic autoimmune rheumatic deseases without adequate humoral response post standard mRNA vaccination. Canadian Institutes of Health Research (CIHR). 995.312 CAD. CO-PI: Gaston De Serres, Ines Colmegna. co-investigators: Bazin R, Dionne M, Hudson M, Bernatsky S, Finzi A, Kaufmann D, Bourré-Tessier J, Flamand L, Libman M, Dieudé M, Goard C, Mendel A.<br>7) Co-Investigator. Co-developing a fLexible cAre delivery model for Rheumatic InflammaTorY diseases. CIHR Planning & Dissemination Grant –ICS (Winter 2022). 20,000 CAD. PI: Diane Lacaille, co-investigators : Bansback N, Barber C, Barnabe C, Bartlett S, Da Costa, Hazlewood G, Hoens A, Koehn C, Li L, Oelke N.Patient Partners: Collins J (APAB), Gervais F (PIRA). Collaborators: Au D, Bardi M, Bessette L, Caron M, Eastwood C, Kur J, MacMullan P, Patrick P, Shojania K<br>8) Co-Investigator. FcgRI as a potential therapeutic target in inflammatory arthritis. Arthritis Society Strategic Operating Grant competition.PI: Marc Pouliot<br>9) Co-Principal Investigator. COVID-19 Vaccine in Immunosuppressed Adults with Autoimmune Diseases. Ministère de la Santé et des Services Sociaux (MSSS). 808,000 CAD.<br>10) Co-Investigator. The UNIversité Laval SARS-CoV-2 variant Research Network (UNICORN). Secrétariat Inter-Conseils (Canada) (CRSH, CRSNG, IRSC). 100,000 CAD [Grant] PI: Louis Flamand, co-investigators: Marc Brisson, Vincent Raymond, Denis Leclerc, Mariana Baz, Paul R. Fortin, Eric Boilard,

			Nicolas Flamand, Marc Pouliot, Ève Dubé, Yannick Doyon.<br>11) Co-Investigator. Safety immUnogenicity of Covid-19 vaCcines in systEmic immunE mediated inflammatory Diseases (SUCCEED). COVID-19 Immunity Task Force. 3,187,905 CAD [Grant] PI: Sasha Bernatsky, co-investigators: Vinod Chandran, Dawn Bowdish, Carol Hitchon, Anne-Claude GINGRAS, Nigil Haroon, Robert Inman, Richard Cook, Gilaad Kaplan, Allison McGeer, Vincent Piguet, Proton Rahman, Mark Silverberg, Tania Watts, Bindee Kuriya, Stephanie Garner, Maggie Larché, John Marshall, Ishac Nazy, Antonio Avina-Zubieta, Cheryl Barnabe, Gilles Boire, Ines Colmegna, Glen Hazelwood, Diane Lacaille, Jessica Widdifield, Karen Colwill, Heidi Wood.<br>12) Co-Principal Investigator. Platelets and neutrophils: the two culprits mediating pain in inflammatory arthritis. Canadian Institutes of Health Research. 910,350 CAD [Grant] PI: Éric Boilard, co-investigators: Clémence Belleannée, Steeve Lacroix<br>13) Principal Investigator. The impact of antimalarial drugs in arthritis patients exposed to SARS-CoV-2-the CoVIRAL project. The Arthritis Society. COVID-20-001. 300,000 CAD [Grant] Co- Principal Investigators: Eric Boilard, Deborah Da Costa<br>14) Co-Principal Investigator. Platelets and neutrophils: The two culprits mediating pain in inflammatory arthritis. The Arthritis Society. 360,000 CAD [Grant] Declined due to overlap with CIHR. PI: Éric Boilard, co-applicants: Yotis Senis, Clémence Belleannée, Steve Lacroix, Louis Bessette<br>15) Collaborator. Impact of the autoimmune anti-LG3 response on Lupus Nephritis. Kidney foundation of Canada 180,000 CAD [Grant] PI: Mélanie Dieudé, Co-applicant: Heloise Cardinal, Collaborators: Eric Boilard, Marie Josee Hebert, Joyce Rauch<br>16) Collaborator. Work disability and function in systematic lupus erythematosus: a national mixed-methods sequential explanatory study. Canadian Initiative for Outcomes in Rheumatology cAre. 117, 098 CAD [Grant] PI: Behdin Nowrouzi-Kia, Zahi Touma<br>17) Principal Investigator. Mapping anti-mitochondrial antibodies in systemic lupus erythematosus. Canadian Institutes of Health Research. 416,280 CAD [Grant] Co-Investigator. Hydroxychloroquine in systemic lupus. Canadian Institutes of Health Research. 342,340 CAD [Grant]
3	Royalties or licenses	__X__None	

4	Consulting fees	__X__None	
5	Payment or honoraria for lectures, presentations, speakers bureaus, manuscript writing or educational events	__X__None	
6	Payment for expert testimony	__X__None	
7	Support for attending meetings and/or travel	__X__None	
8	Patents planned, issued or pending	____None	1) Urinary Biomarkers for SLE and Lupus Nephritis. Applied, Filing Date: 2016. Patents #: PCT/CA2015/051016, United States. Joint Holder Name(s): Joan Wither, Carolina Landolt-Marticorena, Paul Fortin, Paul Boutros, Heather Reich, James Scholey, Carmen Avila-Casado.
			2) Methods and Kits for Diagnosing Systemic Autoimmune Rheumatic Diseases.Patents #: PCT/CA2022/050849. Quebec. Fortin P, Boilard E, Becker Y. V/Réf. : n/a - N/Réf.: 000819-0436
9	Participation on a Data Safety Monitoring Board or Advisory Board	____None	Advisory Board for GSK and Astra-Zeneca.
10	Leadership or fiduciary role in other board, society, committee or advocacy group, paid or unpaid	____None	Vice-Chair of SLICC group (Systemic Lupus International Collaborating Clinics).
			Past-Chair on Executive committee of CaNIOS group (Canadian Network for Improved Outcomes in SLE).
11	Stock or stock options	__X__None	
12	Receipt of equipment, materials, drugs, medical writing, gifts or other services	__X__None	
13	Other financial or non-financial interests	__X__None	

Please place an "X" next to the following statement to indicate your agreement:

_X__ I certify that I have answered every question and have not altered the wording of any of the questions on this form.

16 FEB 2023

ICMJE DISCLOSURE FORM

Date: February 14th 2023
Your Name:_____**Dr Dafna Gladman**_____________________
Manuscript Title : Association between severe non-adherence to hydroxychloroquine and SLE flares, damage, and mortality in 660 patients from the SLICC Inception Cohort
Manuscript number (if known): ar-22-1545.R1

In the interest of transparency, we ask you to disclose all relationships/activities/interests listed below that are related to the content of your manuscript. "Related" means any relation with for-profit or not-for-profit third parties whose interests may be affected by the content of the manuscript. Disclosure represents a commitment to transparency and does not necessarily indicate a bias. If you are in doubt about whether to list a relationship/activity/interest, it is preferable that you do so.

The following questions apply to the author's relationships/activities/interests as they relate to the current manuscript only.

The author's relationships/activities/interests should be defined broadly. For example, if your manuscript pertains to the epidemiology of hypertension, you should declare all relationships with manufacturers of antihypertensive medication, even if that medication is not mentioned in the manuscript.

In item #1 below, report all support for the work reported in this manuscript without time limit. For all other items, the time frame for disclosure is the past 36 months.

		Name all entities with whom you have this relationship or indicate none (add rows as needed)	Specifications/Comments (e.g., if payments were made to you or to your institution)
**Time frame: Since the initial planning of the work**			
1	All support for the present manuscript (e.g., funding, provision of study materials, medical writing, article processing charges, etc.) **No time limit for this item.**	_X__None	
**Time frame: past 36 months**			
2	Grants or contracts from any entity (if not indicated in item #1 above).	_X__None	
3	Royalties or licenses	_X__None	
4	Consulting fees	_X__None	

5	Payment or honoraria for lectures, presentations, speakers bureaus, manuscript writing or educational events	_X__None	
6	Payment for expert testimony	_X__None	
7	Support for attending meetings and/or travel	_X__None	
8	Patents planned, issued or pending	_X__None	
9	Participation on a Data Safety Monitoring Board or Advisory Board	_X__None	
10	Leadership or fiduciary role in other board, society, committee or advocacy group, paid or unpaid	_X__None	
11	Stock or stock options	_X__None	
12	Receipt of equipment, materials, drugs, medical writing, gifts or other services	_X__None	
13	Other financial or non-financial interests	_X__None	

Please place an "X" next to the following statement to indicate your agreement:

__X_ I certify that I have answered every question and have not altered the wording of any of the questions on this form.

ICMJE DISCLOSURE FORM

Date: February 14th 2023
Your Name:_____Ian Bruce_____________________
Manuscript Title : Association between severe non-adherence to hydroxychloroquine and SLE flares, damage, and mortality in 660 patients from the SLICC Inception Cohort
Manuscript number (if known): ar-22-1545.R1

In the interest of transparency, we ask you to disclose all relationships/activities/interests listed below that are related to the content of your manuscript. "Related" means any relation with for-profit or not-for-profit third parties whose interests may be affected by the content of the manuscript. Disclosure represents a commitment to transparency and does not necessarily indicate a bias. If you are in doubt about whether to list a relationship/activity/interest, it is preferable that you do so.

The following questions apply to the author's relationships/activities/interests as they relate to the current manuscript only.

The author's relationships/activities/interests should be defined broadly. For example, if your manuscript pertains to the epidemiology of hypertension, you should declare all relationships with manufacturers of antihypertensive medication, even if that medication is not mentioned in the manuscript.

In item #1 below, report all support for the work reported in this manuscript without time limit. For all other items, the time frame for disclosure is the past 36 months.

		Name all entities with whom you have this relationship or indicate none (add rows as needed)	Specifications/Comments (e.g., if payments were made to you or to your institution)
		**Time frame: Since the initial planning of the work**	
1	All support for the present manuscript (e.g., funding, provision of study materials, medical writing, article processing charges, etc.) **No time limit for this item.**	_X__None	
		**Time frame: past 36 months**	
2	Grants or contracts from any entity (if not indicated in item #1 above).	_X__None	
3	Royalties or licenses	_X__None	
4	Consulting fees	_X__None	

5	Payment or honoraria for lectures, presentations, speakers bureaus, manuscript writing or educational events	_X__None	
6	Payment for expert testimony	_X__None	
7	Support for attending meetings and/or travel	_X__None	
8	Patents planned, issued or pending	_X__None	
9	Participation on a Data Safety Monitoring Board or Advisory Board	_X__None	
10	Leadership or fiduciary role in other board, society, committee or advocacy group, paid or unpaid	_X__None	
11	Stock or stock options	_X__None	
12	Receipt of equipment, materials, drugs, medical writing, gifts or other services	_X__None	
13	Other financial or non-financial interests	_X__None	

Please place an "X" next to the following statement to indicate your agreement:

__X_ I certify that I have answered every question and have not altered the wording of any of the questions on this form.

ICMJE DISCLOSURE FORM

Date: February 14th 2023
Your Name:____Michelle Petri MD MPH_________
Manuscript Title : Association between severe non-adherence to hydroxychloroquine and SLE flares, damage, and mortality in 660 patients from the SLICC Inception Cohort
Manuscript number (if known): ar-22-1545.R1

In the interest of transparency, we ask you to disclose all relationships/activities/interests listed below that are related to the content of your manuscript. "Related" means any relation with for-profit or not-for-profit third parties whose interests may be affected by the content of the manuscript. Disclosure represents a commitment to transparency and does not necessarily indicate a bias. If you are in doubt about whether to list a relationship/activity/interest, it is preferable that you do so.

The following questions apply to the author's relationships/activities/interests as they relate to the current manuscript only.

The author's relationships/activities/interests should be defined broadly. For example, if your manuscript pertains to the epidemiology of hypertension, you should declare all relationships with manufacturers of antihypertensive medication, even if that medication is not mentioned in the manuscript.

In item #1 below, report all support for the work reported in this manuscript without time limit. For all other items, the time frame for disclosure is the past 36 months.

		**Name all entities with whom you have this relationship or indicate none (add rows as needed)**	**Specifications/Comments (e.g., if payments were made to you or to your institution)**
		**Time frame: Since the initial planning of the work**	
1	All support for the present manuscript (e.g., funding, provision of study materials, medical writing, article processing charges, etc.) **No time limit for this item.**	_X___None	
		**Time frame: past 36 months**	
2	Grants or contracts from any entity (if not indicated in item #1 above).	__X__None	
3	Royalties or licenses	__X__None	
4	Consulting fees	__X__None	

5	Payment or honoraria for lectures, presentations, speakers bureaus, manuscript writing or educational events	__X__None	
6	Payment for expert testimony	__X__None	
7	Support for attending meetings and/or travel	_X___None	
8	Patents planned, issued or pending	__X__None	
9	Participation on a Data Safety Monitoring Board or Advisory Board	__X__None	
10	Leadership or fiduciary role in other board, society, committee or advocacy group, paid or unpaid	_X___None	
11	Stock or stock options	__X__None	
12	Receipt of equipment, materials, drugs, medical writing, gifts or other services	__X__None	
13	Other financial or non-financial interests	__X__None	

Please place an "X" next to the following statement to indicate your agreement:

__X_ I certify that I have answered every question and have not altered the wording of any of the questions on this form.

ICMJE DISCLOSURE FORM

Date: February 14th 2023
Your Name:____Ellen Ginzler_________
Manuscript Title : Association between severe non-adherence to hydroxychloroquine and SLE flares, damage, and mortality in 660 patients from the SLICC Inception Cohort
Manuscript number (if known): ar-22-1545.R1

In the interest of transparency, we ask you to disclose all relationships/activities/interests listed below that are related to the content of your manuscript. "Related" means any relation with for-profit or not-for-profit third parties whose interests may be affected by the content of the manuscript. Disclosure represents a commitment to transparency and does not necessarily indicate a bias. If you are in doubt about whether to list a relationship/activity/interest, it is preferable that you do so.

The following questions apply to the author's relationships/activities/interests as they relate to the current manuscript only.

The author's relationships/activities/interests should be defined broadly. For example, if your manuscript pertains to the epidemiology of hypertension, you should declare all relationships with manufacturers of antihypertensive medication, even if that medication is not mentioned in the manuscript.

In item #1 below, report all support for the work reported in this manuscript without time limit. For all other items, the time frame for disclosure is the past 36 months.

		**Name all entities with whom you have this relationship or indicate none (add rows as needed)**	**Specifications/Comments (e.g., if payments were made to you or to your institution)**
		**Time frame: Since the initial planning of the work**	
1	All support for the present manuscript (e.g., funding, provision of study materials, medical writing, article processing charges, etc.) **No time limit for this item.**	_X___None	
		**Time frame: past 36 months**	
2	Grants or contracts from any entity (if not indicated in item #1 above).	__X__None	
3	Royalties or licenses	__X__None	
4	Consulting fees	__X__None	

5	Payment or honoraria for lectures, presentations, speakers bureaus, manuscript writing or educational events	__X__None	
6	Payment for expert testimony	__X__None	
7	Support for attending meetings and/or travel	_X___None	
8	Patents planned, issued or pending	__X__None	
9	Participation on a Data Safety Monitoring Board or Advisory Board	__X__None	
10	Leadership or fiduciary role in other board, society, committee or advocacy group, paid or unpaid	_X___None	
11	Stock or stock options	__X__None	
12	Receipt of equipment, materials, drugs, medical writing, gifts or other services	__X__None	
13	Other financial or non-financial interests	__X__None	

Please place an "X" next to the following statement to indicate your agreement:

__X_ I certify that I have answered every question and have not altered the wording of any of the questions on this form.

ICMJE DISCLOSURE FORM

Date: February 14th 2023
Your Name:____Mary Anne Dooley_________
Manuscript Title : Association between severe non-adherence to hydroxychloroquine and SLE flares, damage, and mortality in 660 patients from the SLICC Inception Cohort
Manuscript number (if known): ar-22-1545.R1

In the interest of transparency, we ask you to disclose all relationships/activities/interests listed below that are related to the content of your manuscript. "Related" means any relation with for-profit or not-for-profit third parties whose interests may be affected by the content of the manuscript. Disclosure represents a commitment to transparency and does not necessarily indicate a bias. If you are in doubt about whether to list a relationship/activity/interest, it is preferable that you do so.

The following questions apply to the author's relationships/activities/interests as they relate to the current manuscript only.

The author's relationships/activities/interests should be defined broadly. For example, if your manuscript pertains to the epidemiology of hypertension, you should declare all relationships with manufacturers of antihypertensive medication, even if that medication is not mentioned in the manuscript.

In item #1 below, report all support for the work reported in this manuscript without time limit. For all other items, the time frame for disclosure is the past 36 months.

		**Name all entities with whom you have this relationship or indicate none (add rows as needed)**	**Specifications/Comments (e.g., if payments were made to you or to your institution)**
**Time frame: Since the initial planning of the work**			
1	All support for the present manuscript (e.g., funding, provision of study materials, medical writing, article processing charges, etc.) **No time limit for this item.**	_X___None	
**Time frame: past 36 months**			
2	Grants or contracts from any entity (if not indicated in item #1 above).	__X__None	
3	Royalties or licenses	__X__None	
4	Consulting fees	__X__None	

5	Payment or honoraria for lectures, presentations, speakers bureaus, manuscript writing or educational events	__X__None	
6	Payment for expert testimony	__X__None	
7	Support for attending meetings and/or travel	_X___None	
8	Patents planned, issued or pending	__X__None	
9	Participation on a Data Safety Monitoring Board or Advisory Board	__X__None	
10	Leadership or fiduciary role in other board, society, committee or advocacy group, paid or unpaid	_X___None	
11	Stock or stock options	__X__None	
12	Receipt of equipment, materials, drugs, medical writing, gifts or other services	__X__None	
13	Other financial or non-financial interests	__X__None	

Please place an "X" next to the following statement to indicate your agreement:

__X_ I certify that I have answered every question and have not altered the wording of any of the questions on this form.

ICMJE DISCLOSURE FORM

Date: February 14th 2023
Your Name:_____Rosalind Ramsey-Goldman_____________________
Manuscript Title : Association between severe non-adherence to hydroxychloroquine and SLE flares, damage, and mortality in 660 patients from the SLICC Inception Cohort
Manuscript number (if known): ar-22-1545.R1

In the interest of transparency, we ask you to disclose all relationships/activities/interests listed below that are related to the content of your manuscript. "Related" means any relation with for-profit or not-for-profit third parties whose interests may be affected by the content of the manuscript. Disclosure represents a commitment to transparency and does not necessarily indicate a bias. If you are in doubt about whether to list a relationship/activity/interest, it is preferable that you do so.

The following questions apply to the author's relationships/activities/interests as they relate to the <u>current manuscript only</u>.

The author's relationships/activities/interests should be <u>defined broadly</u>. For example, if your manuscript pertains to the epidemiology of hypertension, you should declare all relationships with manufacturers of antihypertensive medication, even if that medication is not mentioned in the manuscript.

In item #1 below, report all support for the work reported in this manuscript without time limit. For all other items, the time frame for disclosure is the past 36 months.

		**Name all entities with whom you have this relationship or indicate none (add rows as needed)**	**Specifications/Comments (e.g., if payments were made to you or to your institution)**
**Time frame: Since the initial planning of the work**			
1	All support for the present manuscript (e.g., funding, provision of study materials, medical writing, article processing charges, etc.) **No time limit for this item.**	_x___None	
**Time frame: past 36 months**			
2	Grants or contracts from any entity (if not indicated in item #1 above).	__x__None	
3	Royalties or licenses	__x__None	

4	Consulting fees	Exagen, Inc.; Biogen; Cabaletta; Fisher Scientific; Horizon	
5	Payment or honoraria for lectures, presentations, speakers bureaus, manuscript writing or educational events	AstraZeneca	
6	Payment for expert testimony	_x___None	
7	Support for attending meetings and/or travel	__x__None	
8	Patents planned, issued or pending	__x__None	
9	Participation on a Data Safety Monitoring Board or Advisory Board	___x_None	
10	Leadership or fiduciary role in other board, society, committee or advocacy group, paid or unpaid	___x_None	
11	Stock or stock options	__x__None	
12	Receipt of equipment, materials, drugs, medical writing, gifts or other services	_x___None	
13	Other financial or non-financial interests	_x___None	

Please place an "X" next to the following statement to indicate your agreement:

___x I certify that I have answered every question and have not altered the wording of any of the questions on this form.

ICMJE DISCLOSURE FORM

Date: February 14th 2023
Your Name:____Susan Manzi_________
Manuscript Title : Association between severe non-adherence to hydroxychloroquine and SLE flares, damage, and mortality in 660 patients from the SLICC Inception Cohort
Manuscript number (if known): ar-22-1545.R1

In the interest of transparency, we ask you to disclose all relationships/activities/interests listed below that are related to the content of your manuscript. "Related" means any relation with for-profit or not-for-profit third parties whose interests may be affected by the content of the manuscript. Disclosure represents a commitment to transparency and does not necessarily indicate a bias. If you are in doubt about whether to list a relationship/activity/interest, it is preferable that you do so.

The following questions apply to the author's relationships/activities/interests as they relate to the current manuscript only.

The author's relationships/activities/interests should be defined broadly. For example, if your manuscript pertains to the epidemiology of hypertension, you should declare all relationships with manufacturers of antihypertensive medication, even if that medication is not mentioned in the manuscript.

In item #1 below, report all support for the work reported in this manuscript without time limit. For all other items, the time frame for disclosure is the past 36 months.

		**Name all entities with whom you have this relationship or indicate none (add rows as needed)**	**Specifications/Comments (e.g., if payments were made to you or to your institution)**
		**Time frame: Since the initial planning of the work**	
1	All support for the present manuscript (e.g., funding, provision of study materials, medical writing, article processing charges, etc.) **No time limit for this item.**	_X___None	
		**Time frame: past 36 months**	
2	Grants or contracts from any entity (if not indicated in item #1 above).	__X__None	
3	Royalties or licenses	__X__None	
4	Consulting fees	__X__None	

5	Payment or honoraria for lectures, presentations, speakers bureaus, manuscript writing or educational events	__X__None	
6	Payment for expert testimony	__X__None	
7	Support for attending meetings and/or travel	_X___None	
8	Patents planned, issued or pending	__X__None	
9	Participation on a Data Safety Monitoring Board or Advisory Board	__X__None	
10	Leadership or fiduciary role in other board, society, committee or advocacy group, paid or unpaid	_X___None	
11	Stock or stock options	__X__None	
12	Receipt of equipment, materials, drugs, medical writing, gifts or other services	__X__None	
13	Other financial or non-financial interests	__X__None	

Please place an "X" next to the following statement to indicate your agreement:

__X_ I certify that I have answered every question and have not altered the wording of any of the questions on this form.

ICMJE DISCLOSURE FORM

Date: February 14th 2023
Your Name:____Andreas Jönsen_________
Manuscript Title : Association between severe non-adherence to hydroxychloroquine and SLE flares, damage, and mortality in 660 patients from the SLICC Inception Cohort
Manuscript number (if known): ar-22-1545.R1

In the interest of transparency, we ask you to disclose all relationships/activities/interests listed below that are related to the content of your manuscript. "Related" means any relation with for-profit or not-for-profit third parties whose interests may be affected by the content of the manuscript. Disclosure represents a commitment to transparency and does not necessarily indicate a bias. If you are in doubt about whether to list a relationship/activity/interest, it is preferable that you do so.

The following questions apply to the author's relationships/activities/interests as they relate to the current manuscript only.

The author's relationships/activities/interests should be defined broadly. For example, if your manuscript pertains to the epidemiology of hypertension, you should declare all relationships with manufacturers of antihypertensive medication, even if that medication is not mentioned in the manuscript.

In item #1 below, report all support for the work reported in this manuscript without time limit. For all other items, the time frame for disclosure is the past 36 months.

		**Name all entities with whom you have this relationship or indicate none (add rows as needed)**	**Specifications/Comments (e.g., if payments were made to you or to your institution)**
		**Time frame: Since the initial planning of the work**	
1	All support for the present manuscript (e.g., funding, provision of study materials, medical writing, article processing charges, etc.) **No time limit for this item.**	_X___None	
		**Time frame: past 36 months**	
2	Grants or contracts from any entity (if not indicated in item #1 above).	__X__None	
3	Royalties or licenses	__X__None	
4	Consulting fees	__X__None	

5	Payment or honoraria for lectures, presentations, speakers bureaus, manuscript writing or educational events	__X__None	
6	Payment for expert testimony	__X__None	
7	Support for attending meetings and/or travel	_X___None	
8	Patents planned, issued or pending	__X__None	
9	Participation on a Data Safety Monitoring Board or Advisory Board	__X__None	
10	Leadership or fiduciary role in other board, society, committee or advocacy group, paid or unpaid	_X___None	
11	Stock or stock options	__X__None	
12	Receipt of equipment, materials, drugs, medical writing, gifts or other services	__X__None	
13	Other financial or non-financial interests	__X__None	

Please place an "X" next to the following statement to indicate your agreement:

__X_ I certify that I have answered every question and have not altered the wording of any of the questions on this form.

ICMJE DISCLOSURE FORM

Date: February 14th 2023
Your Name: Graciela S. Alarcón__________________________
Manuscript Title : Association between severe non-adherence to hydroxychloroquine and SLE flares, damage, and mortality in 660 patients from the SLICC Inception Cohort
Manuscript number (if known): ar-22-1545.R1

In the interest of transparency, we ask you to disclose all relationships/activities/interests listed below that are related to the content of your manuscript. "Related" means any relation with for-profit or not-for-profit third parties whose interests may be affected by the content of the manuscript. Disclosure represents a commitment to transparency and does not necessarily indicate a bias. If you are in doubt about whether to list a relationship/activity/interest, it is preferable that you do so.

The following questions apply to the author's relationships/activities/interests as they relate to the current manuscript only.

The author's relationships/activities/interests should be defined broadly. For example, if your manuscript pertains to the epidemiology of hypertension, you should declare all relationships with manufacturers of antihypertensive medication, even if that medication is not mentioned in the manuscript.

In item #1 below, report all support for the work reported in this manuscript without time limit. For all other items, the time frame for disclosure is the past 36 months.

		**Name all entities with whom you have this relationship or indicate none (add rows as needed)**	**Specifications/Comments (e.g., if payments were made to you or to your institution)**
		**Time frame: Since the initial planning of the work**	
1	All support for the present manuscript (e.g., funding, provision of study materials, medical writing, article processing charges, etc.) **No time limit for this item.**	_X__None	
		**Time frame: past 36 months**	
2	Grants or contracts from any entity (if not indicated in item #1 above).	_X__None	
3	Royalties or licenses	_X__None	
4	Consulting fees	_X__None	

5	Payment or honoraria for lectures, presentations, speakers bureaus, manuscript writing or educational events	_X__None	
6	Payment for expert testimony	_X__None	
7	Support for attending meetings and/or travel	_X__None	
8	Patents planned, issued or pending	_X__None	
9	Participation on a Data Safety Monitoring Board or Advisory Board	_X__None	
10	Leadership or fiduciary role in other board, society, committee or advocacy group, paid or unpaid	_X__None	
11	Stock or stock options	_X__None	
12	Receipt of equipment, materials, drugs, medical writing, gifts or other services	_X__None	
13	Other financial or non-financial interests	_X__None	

Please place an "X" next to the following statement to indicate your agreement:

X certify that I have answered every question and have not altered the wording of any of the questions on this form.

ICMJE DISCLOSURE FORM

Date: February 14th 2023
Your Name:____Ronald van Vollenhoven______________________
Manuscript Title : Association between severe non-adherence to hydroxychloroquine and SLE flares, damage, and mortality in 660 patients from the SLICC Inception Cohort
Manuscript number (if known): ar-22-1545.R1

In the interest of transparency, we ask you to disclose all relationships/activities/interests listed below that are related to the content of your manuscript. "Related" means any relation with for-profit or not-for-profit third parties whose interests may be affected by the content of the manuscript. Disclosure represents a commitment to transparency and does not necessarily indicate a bias. If you are in doubt about whether to list a relationship/activity/interest, it is preferable that you do so.

The following questions apply to the author's relationships/activities/interests as they relate to the current manuscript only.

The author's relationships/activities/interests should be defined broadly. For example, if your manuscript pertains to the epidemiology of hypertension, you should declare all relationships with manufacturers of antihypertensive medication, even if that medication is not mentioned in the manuscript.

In item #1 below, report all support for the work reported in this manuscript without time limit. For all other items, the time frame for disclosure is the past 36 months.

		**Name all entities with whom you have this relationship or indicate none (add rows as needed)**	**Specifications/Comments (e.g., if payments were made to you or to your institution)**
**Time frame: Since the initial planning of the work**			
1	All support for the present manuscript (e.g., funding, provision of study materials, medical writing, article processing charges, etc.) **No time limit for this item.**	_X__None	
**Time frame: past 36 months**			
2	Grants or contracts from any entity (if not indicated in item #1 above).	BMS, GSK, Lilly, UCB, Pfizer, Roche	
3	Royalties or licenses	_X__None	

4	Consulting fees	AbbVie, AstraZeneca, Biogen, BMS, Galapagos, Janssen, Pfizer, Sanofi, UCB	
5	Payment or honoraria for lectures, presentations, speakers bureaus, manuscript writing or educational events	AbbVie, Galapagos, Janssen, Pfizer, UCB	
6	Payment for expert testimony	_X__None	
7	Support for attending meetings and/or travel	_X__None	
8	Patents planned, issued or pending	_X__None	
9	Participation on a Data Safety Monitoring Board or Advisory Board	AbbVie, AstraZeneca, Biogen, BMS, Galapagos, Janssen, Pfizer, Sanofi, UCB	
10	Leadership or fiduciary role in other board, society, committee or advocacy group, paid or unpaid	_X__None	
11	Stock or stock options	_X__None	
12	Receipt of equipment, materials, drugs, medical writing, gifts or other services	_X__None	
13	Other financial or non-financial interests	_X__None	

Please place an "X" next to the following statement to indicate your agreement:

__x_ I certify that I have answered every question and have not altered the wording of any of the questions on this form.

ICMJE DISCLOSURE FORM

Date: February 14th 2023
Your Name:___**Cynthia Aranow**__________
Manuscript Title : Association between severe non-adherence to hydroxychloroquine and SLE flares, damage, and mortality in 660 patients from the SLICC Inception Cohort
Manuscript number (if known): ar-22-1545.R1

In the interest of transparency, we ask you to disclose all relationships/activities/interests listed below that are related to the content of your manuscript. "Related" means any relation with for-profit or not-for-profit third parties whose interests may be affected by the content of the manuscript. Disclosure represents a commitment to transparency and does not necessarily indicate a bias. If you are in doubt about whether to list a relationship/activity/interest, it is preferable that you do so.

The following questions apply to the author's relationships/activities/interests as they relate to the current manuscript only.

The author's relationships/activities/interests should be defined broadly. For example, if your manuscript pertains to the epidemiology of hypertension, you should declare all relationships with manufacturers of antihypertensive medication, even if that medication is not mentioned in the manuscript.

In item #1 below, report all support for the work reported in this manuscript without time limit. For all other items, the time frame for disclosure is the past 36 months.

		Name all entities with whom you have this relationship or indicate none (add rows as needed)	Specifications/Comments (e.g., if payments were made to you or to your institution)
		**Time frame: Since the initial planning of the work**	
1	All support for the present manuscript (e.g., funding, provision of study materials, medical writing, article processing charges, etc.) **No time limit for this item.**	____None	
		**Time frame: past 36 months**	
2	Grants or contracts from any entity (if not indicated in item #1 above).	____None	
3	Royalties or licenses	____None	
4	Consulting fees	____None	

5	Payment or honoraria for lectures, presentations, speakers bureaus, manuscript writing or educational events	____None	
6	Payment for expert testimony	____None	
7	Support for attending meetings and/or travel	____None	
8	Patents planned, issued or pending	____None	
9	Participation on a Data Safety Monitoring Board or Advisory Board	____None	
10	Leadership or fiduciary role in other board, society, committee or advocacy group, paid or unpaid	____None	
11	Stock or stock options	____None	
12	Receipt of equipment, materials, drugs, medical writing, gifts or other services	____None	
13	Other financial or non-financial interests	____None	

Please place an "X" next to the following statement to indicate your agreement:

_x_ **I certify that I have answered every question and have not altered the wording of any of the questions on this form.**

[illegible] 2/14/23

ICMJE DISCLOSURE FORM

Date: February 14th 2023
Your Name:___**Veronique Le Guern**______________________
Manuscript Title : Association between severe non-adherence to hydroxychloroquine and SLE flares, damage, and mortality in 660 patients from the SLICC Inception Cohort
Manuscript number (if known): ar-22-1545.R1

In the interest of transparency, we ask you to disclose all relationships/activities/interests listed below that are related to the content of your manuscript. "Related" means any relation with for-profit or not-for-profit third parties whose interests may be affected by the content of the manuscript. Disclosure represents a commitment to transparency and does not necessarily indicate a bias. If you are in doubt about whether to list a relationship/activity/interest, it is preferable that you do so.

The following questions apply to the author's relationships/activities/interests as they relate to the current manuscript only.

The author's relationships/activities/interests should be defined broadly. For example, if your manuscript pertains to the epidemiology of hypertension, you should declare all relationships with manufacturers of antihypertensive medication, even if that medication is not mentioned in the manuscript.

In item #1 below, report all support for the work reported in this manuscript without time limit. For all other items, the time frame for disclosure is the past 36 months.

		**Name all entities with whom you have this relationship or indicate none (add rows as needed)**	**Specifications/Comments (e.g., if payments were made to you or to your institution)**
		Time frame: Since the initial planning of the work	
1	All support for the present manuscript (e.g., funding, provision of study materials, medical writing, article processing charges, etc.) **No time limit for this item.**	None	
		Time frame: past 36 months	
2	Grants or contracts from any entity (if not indicated in item #1 above).	None	
3	Royalties or licenses	None	
4	Consulting fees	None	

5	Payment or honoraria for lectures, presentations, speakers bureaus, manuscript writing or educational events	____None	
		Bristol Meyer Squibb	presentations
6	Payment for expert testimony	__None (circled)	
7	Support for attending meetings and/or travel	____None	
		Novartis	Meeting and travel
		Astra Zeneca	Meeting and travel
8	Patents planned, issued or pending	__None (circled)	
9	Participation on a Data Safety Monitoring Board or Advisory Board	____None	
		Novartis	Advisory Board
10	Leadership or fiduciary role in other board, society, committee or advocacy group, paid or unpaid	__None (circled)	
11	Stock or stock options	__None (circled)	
12	Receipt of equipment, materials, drugs, medical writing, gifts or other services	__None (circled)	
13	Other financial or non-financial interests	__None (circled)	

Please place an "X" next to the following statement to indicate your agreement:

X **I certify that I have answered every question and have not altered the wording of any of the questions on this form.**

ICMJE DISCLOSURE FORM

Date: February 14th 2023
Your Name:_____**Meggan Mackay**__________________
Manuscript Title : Association between severe non-adherence to hydroxychloroquine and SLE flares, damage, and mortality in 660 patients from the SLICC Inception Cohort
Manuscript number (if known): ar-22-1545.R1

In the interest of transparency, we ask you to disclose all relationships/activities/interests listed below that are related to the content of your manuscript. "Related" means any relation with for-profit or not-for-profit third parties whose interests may be affected by the content of the manuscript. Disclosure represents a commitment to transparency and does not necessarily indicate a bias. If you are in doubt about whether to list a relationship/activity/interest, it is preferable that you do so.

The following questions apply to the author's relationships/activities/interests as they relate to the current manuscript only.

The author's relationships/activities/interests should be defined broadly. For example, if your manuscript pertains to the epidemiology of hypertension, you should declare all relationships with manufacturers of antihypertensive medication, even if that medication is not mentioned in the manuscript.

In item #1 below, report all support for the work reported in this manuscript without time limit. For all other items, the time frame for disclosure is the past 36 months.

		**Name all entities with whom you have this relationship or indicate none (add rows as needed)**	**Specifications/Comments (e.g., if payments were made to you or to your institution)**
**Time frame: Since the initial planning of the work**			
1	All support for the present manuscript (e.g., funding, provision of study materials, medical writing, article processing charges, etc.) **No time limit for this item.**	___X_None	
**Time frame: past 36 months**			
2	Grants or contracts from any entity (if not indicated in item #1 above).	__X__None	
3	Royalties or licenses	__X__None	
4	Consulting fees	___X_None	

5	Payment or honoraria for lectures, presentations, speakers bureaus, manuscript writing or educational events	___X_None	
6	Payment for expert testimony	__X__None	
7	Support for attending meetings and/or travel	__X__None	
8	Patents planned, issued or pending	__X__None	
9	Participation on a Data Safety Monitoring Board or Advisory Board	__X__None	
10	Leadership or fiduciary role in other board, society, committee or advocacy group, paid or unpaid	__X__None	
11	Stock or stock options	__X__None	
12	Receipt of equipment, materials, drugs, medical writing, gifts or other services	_X___None	
13	Other financial or non-financial interests	_X___None	

Please place an "X" next to the following statement to indicate your agreement:

__X_ I certify that I have answered every question and have not altered the wording of any of the questions on this form.

ICMJE DISCLOSURE FORM

Date: February 14th 2023
Your Name: GUILLERMO RUIZ-IRASTORZA
Manuscript Title : Association between severe non-adherence to hydroxychloroquine and SLE flares, damage, and mortality in 660 patients from the SLICC Inception Cohort
Manuscript number (if known): ar-22-1545.R1

In the interest of transparency, we ask you to disclose all relationships/activities/interests listed below that are related to the content of your manuscript. "Related" means any relation with for-profit or not-for-profit third parties whose interests may be affected by the content of the manuscript. Disclosure represents a commitment to transparency and does not necessarily indicate a bias. If you are in doubt about whether to list a relationship/activity/interest, it is preferable that you do so.

The following questions apply to the author's relationships/activities/interests as they relate to the current manuscript only.

The author's relationships/activities/interests should be defined broadly. For example, if your manuscript pertains to the epidemiology of hypertension, you should declare all relationships with manufacturers of antihypertensive medication, even if that medication is not mentioned in the manuscript.

In item #1 below, report all support for the work reported in this manuscript without time limit. For all other items, the time frame for disclosure is the past 36 months.

		Name all entities with whom you have this relationship or indicate none (add rows as needed)	Specifications/Comments (e.g., if payments were made to you or to your institution)
		**Time frame: Since the initial planning of the work**	
1	All support for the present manuscript (e.g., funding, provision of study materials, medical writing, article processing charges, etc.) **No time limit for this item.**	_X__None	
		**Time frame: past 36 months**	
2	Grants or contracts from any entity (if not indicated in item #1 above).	_X__None	
3	Royalties or licenses	_X__None	
4	Consulting fees	_X__None	

5	Payment or honoraria for lectures, presentations, speakers bureaus, manuscript writing or educational events	_X__None	
6	Payment for expert testimony	_X__None	
7	Support for attending meetings and/or travel	_X__None	
8	Patents planned, issued or pending	_X__None	
9	Participation on a Data Safety Monitoring Board or Advisory Board	_X__None	
10	Leadership or fiduciary role in other board, society, committee or advocacy group, paid or unpaid	_X__None	
11	Stock or stock options	_X__None	
12	Receipt of equipment, materials, drugs, medical writing, gifts or other services	_X__None	
13	Other financial or non-financial interests	_X__None	

Please place an "X" next to the following statement to indicate your agreement:

X I certify that I have answered every question and have not altered the wording of any of the questions on this form.

ICMJE DISCLOSURE FORM

Date: February 14th 2023
Your Name:____S. Sam Lim______________________
Manuscript Title : Association between severe non-adherence to hydroxychloroquine and SLE flares, damage, and mortality in 660 patients from the SLICC Inception Cohort
Manuscript number (if known): ar-22-1545.R1

In the interest of transparency, we ask you to disclose all relationships/activities/interests listed below that are related to the content of your manuscript. "Related" means any relation with for-profit or not-for-profit third parties whose interests may be affected by the content of the manuscript. Disclosure represents a commitment to transparency and does not necessarily indicate a bias. If you are in doubt about whether to list a relationship/activity/interest, it is preferable that you do so.

The following questions apply to the author's relationships/activities/interests as they relate to the current manuscript only.

The author's relationships/activities/interests should be defined broadly. For example, if your manuscript pertains to the epidemiology of hypertension, you should declare all relationships with manufacturers of antihypertensive medication, even if that medication is not mentioned in the manuscript.

In item #1 below, report all support for the work reported in this manuscript without time limit. For all other items, the time frame for disclosure is the past 36 months.

		**Name all entities with whom you have this relationship or indicate none (add rows as needed)**	**Specifications/Comments (e.g., if payments were made to you or to your institution)**
		**Time frame: Since the initial planning of the work**	
1	All support for the present manuscript (e.g., funding, provision of study materials, medical writing, article processing charges, etc.) **No time limit for this item.**	_X__None	
		**Time frame: past 36 months**	
2	Grants or contracts from any entity (if not indicated in item #1 above).	_X__None	
3	Royalties or licenses	_X__None	
4	Consulting fees	_X__None	

5	Payment or honoraria for lectures, presentations, speakers bureaus, manuscript writing or educational events	_X__None	
6	Payment for expert testimony	_X__None	
7	Support for attending meetings and/or travel	_X__None	
8	Patents planned, issued or pending	_X__None	
9	Participation on a Data Safety Monitoring Board or Advisory Board	_X__None	
10	Leadership or fiduciary role in other board, society, committee or advocacy group, paid or unpaid	_X__None	
11	Stock or stock options	_X__None	
12	Receipt of equipment, materials, drugs, medical writing, gifts or other services	_X__None	
13	Other financial or non-financial interests	_X__None	

Please place an "X" next to the following statement to indicate your agreement:

__X_ I certify that I have answered every question and have not altered the wording of any of the questions on this form.

ICMJE DISCLOSURE FORM

Date: February 14th 2023
Your Name: MURAT INANC
Manuscript Title : Association between severe non-adherence to hydroxychloroquine and SLE flares, damage, and mortality in 660 patients from the SLICC Inception Cohort
Manuscript number (if known): ar-22-1545.R1

In the interest of transparency, we ask you to disclose all relationships/activities/interests listed below that are related to the content of your manuscript. "Related" means any relation with for-profit or not-for-profit third parties whose interests may be affected by the content of the manuscript. Disclosure represents a commitment to transparency and does not necessarily indicate a bias. If you are in doubt about whether to list a relationship/activity/interest, it is preferable that you do so.

The following questions apply to the author's relationships/activities/interests as they relate to the current manuscript only.

The author's relationships/activities/interests should be defined broadly. For example, if your manuscript pertains to the epidemiology of hypertension, you should declare all relationships with manufacturers of antihypertensive medication, even if that medication is not mentioned in the manuscript.

In item #1 below, report all support for the work reported in this manuscript without time limit. For all other items, the time frame for disclosure is the past 36 months.

		Name all entities with whom you have this relationship or indicate none (add rows as needed)	Specifications/Comments (e.g., if payments were made to you or to your institution)
		**Time frame: Since the initial planning of the work**	
1	All support for the present manuscript (e.g., funding, provision of study materials, medical writing, article processing charges, etc.) **No time limit for this item.**	✓ None	
		**Time frame: past 36 months**	
2	Grants or contracts from any entity (if not indicated in item #1 above).	✓ None	
3	Royalties or licenses	✓ None	
4	Consulting fees	✓ None	

5	Payment or honoraria for lectures, presentations, speakers bureaus, manuscript writing or educational events	None	
6	Payment for expert testimony	None	
7	Support for attending meetings and/or travel	None	
8	Patents planned, issued or pending	None	
9	Participation on a Data Safety Monitoring Board or Advisory Board	None	
10	Leadership or fiduciary role in other board, society, committee or advocacy group, paid or unpaid	None	
11	Stock or stock options	None	
12	Receipt of equipment, materials, drugs, medical writing, gifts or other services	None	
13	Other financial or non-financial interests	None	

Please place an "X" next to the following statement to indicate your agreement:

X **I certify that I have answered every question and have not altered the wording of any of the questions on this form.**

Murat Inanc

ICMJE DISCLOSURE FORM

Date: February 14th 2023
Your Name:____Kenneth Kalunian______________________
Manuscript Title : Association between severe non-adherence to hydroxychloroquine and SLE flares, damage, and mortality in 660 patients from the SLICC Inception Cohort
Manuscript number (if known): ar-22-1545.R1

In the interest of transparency, we ask you to disclose all relationships/activities/interests listed below that are related to the content of your manuscript. "Related" means any relation with for-profit or not-for-profit third parties whose interests may be affected by the content of the manuscript. Disclosure represents a commitment to transparency and does not necessarily indicate a bias. If you are in doubt about whether to list a relationship/activity/interest, it is preferable that you do so.

The following questions apply to the author's relationships/activities/interests as they relate to the current manuscript only.

The author's relationships/activities/interests should be defined broadly. For example, if your manuscript pertains to the epidemiology of hypertension, you should declare all relationships with manufacturers of antihypertensive medication, even if that medication is not mentioned in the manuscript.

In item #1 below, report all support for the work reported in this manuscript without time limit. For all other items, the time frame for disclosure is the past 36 months.

		**Name all entities with whom you have this relationship or indicate none (add rows as needed)**	**Specifications/Comments (e.g., if payments were made to you or to your institution)**
**Time frame: Since the initial planning of the work**			
1	All support for the present manuscript (e.g., funding, provision of study materials, medical writing, article processing charges, etc.) **No time limit for this item.**	_X__None	
**Time frame: past 36 months**			
2	Grants or contracts from any entity (if not indicated in item #1 above).	_X__None	
3	Royalties or licenses	_X__None	
4	Consulting fees	_X__None	

5	Payment or honoraria for lectures, presentations, speakers bureaus, manuscript writing or educational events	_X__None	
6	Payment for expert testimony	_X__None	
7	Support for attending meetings and/or travel	_X__None	
8	Patents planned, issued or pending	_X__None	
9	Participation on a Data Safety Monitoring Board or Advisory Board	_X__None	
10	Leadership or fiduciary role in other board, society, committee or advocacy group, paid or unpaid	_X__None	
11	Stock or stock options	_X__None	
12	Receipt of equipment, materials, drugs, medical writing, gifts or other services	_X__None	
13	Other financial or non-financial interests	_X__None	

Please place an "X" next to the following statement to indicate your agreement:

__X_ I certify that I have answered every question and have not altered the wording of any of the questions on this form.

ICMJE DISCLOSURE FORM

Date: February 14th 2023
Your Name: Søren Jacobsen
Manuscript Title : Association between severe non-adherence to hydroxychloroquine and SLE flares, damage, and mortality in 660 patients from the SLICC Inception Cohort
Manuscript number (if known): ar-22-1545.R1

In the interest of transparency, we ask you to disclose all relationships/activities/interests listed below that are related to the content of your manuscript. "Related" means any relation with for-profit or not-for-profit third parties whose interests may be affected by the content of the manuscript. Disclosure represents a commitment to transparency and does not necessarily indicate a bias. If you are in doubt about whether to list a relationship/activity/interest, it is preferable that you do so.

The following questions apply to the author's relationships/activities/interests as they relate to the current manuscript only.

The author's relationships/activities/interests should be defined broadly. For example, if your manuscript pertains to the epidemiology of hypertension, you should declare all relationships with manufacturers of antihypertensive medication, even if that medication is not mentioned in the manuscript.

In item #1 below, report all support for the work reported in this manuscript without time limit. For all other items, the time frame for disclosure is the past 36 months.

		**Name all entities with whom you have this relationship or indicate none (add rows as needed)**	**Specifications/Comments (e.g., if payments were made to you or to your institution)**
		**Time frame: Since the initial planning of the work**	
1	All support for the present manuscript (e.g., funding, provision of study materials, medical writing, article processing charges, etc.) **No time limit for this item.**	_X__None	
		**Time frame: past 36 months**	
2	Grants or contracts from any entity (if not indicated in item #1 above).	_X__None	
3	Royalties or licenses	_X__None	
4	Consulting fees	_X__None	

5	Payment or honoraria for lectures, presentations, speakers bureaus, manuscript writing or educational events	_X__None	
6	Payment for expert testimony	_X__None	
7	Support for attending meetings and/or travel	_X__None	
8	Patents planned, issued or pending	_X__None	
9	Participation on a Data Safety Monitoring Board or Advisory Board	_X__None	
10	Leadership or fiduciary role in other board, society, committee or advocacy group, paid or unpaid	_X__None	
11	Stock or stock options	_X__None	
12	Receipt of equipment, materials, drugs, medical writing, gifts or other services	_X__None	
13	Other financial or non-financial interests	_X__None	

Please place an "X" next to the following statement to indicate your agreement:

_X_ I certify that I have answered every question and have not altered the wording of any of the questions on this form.

ICMJE DISCLOSURE FORM

Date: February 14th 2023
Your Name:_______**Christine A Peschken**___________________
Manuscript Title : Association between severe non-adherence to hydroxychloroquine and SLE flares, damage, and mortality in 660 patients from the SLICC Inception Cohort
Manuscript number (if known): ar-22-1545.R1

In the interest of transparency, we ask you to disclose all relationships/activities/interests listed below that are related to the content of your manuscript. "Related" means any relation with for-profit or not-for-profit third parties whose interests may be affected by the content of the manuscript. Disclosure represents a commitment to transparency and does not necessarily indicate a bias. If you are in doubt about whether to list a relationship/activity/interest, it is preferable that you do so.

The following questions apply to the author's relationships/activities/interests as they relate to the current manuscript only.

The author's relationships/activities/interests should be defined broadly. For example, if your manuscript pertains to the epidemiology of hypertension, you should declare all relationships with manufacturers of antihypertensive medication, even if that medication is not mentioned in the manuscript.

In item #1 below, report all support for the work reported in this manuscript without time limit. For all other items, the time frame for disclosure is the past 36 months.

		Name all entities with whom you have this relationship or indicate none (add rows as needed)	Specifications/Comments (e.g., if payments were made to you or to your institution)
		**Time frame: Since the initial planning of the work**	
1	All support for the present manuscript (e.g., funding, provision of study materials, medical writing, article processing charges, etc.) **No time limit for this item.**	_X__None	
		**Time frame: past 36 months**	
2	Grants or contracts from any entity (if not indicated in item #1 above).	_X__None	
3	Royalties or licenses	_X__None	
4	Consulting fees	_X__None	

5	Payment or honoraria for lectures, presentations, speakers bureaus, manuscript writing or educational events	_X__None	
6	Payment for expert testimony	_X__None	
7	Support for attending meetings and/or travel	_X__None	
8	Patents planned, issued or pending	_X__None	
9	Participation on a Data Safety Monitoring Board or Advisory Board	_X__None	
10	Leadership or fiduciary role in other board, society, committee or advocacy group, paid or unpaid	_X__None	
11	Stock or stock options	_X__None	
12	Receipt of equipment, materials, drugs, medical writing, gifts or other services	_X__None	
13	Other financial or non-financial interests	_X__None	

Please place an "X" next to the following statement to indicate your agreement:

_X__ I certify that I have answered every question and have not altered the wording of any of the questions on this form.

ICMJE DISCLOSURE FORM

Date: February 14th 2023
Your Name:_____Diane L. Kamen, MD, MSCR_____________________
Manuscript Title : Association between severe non-adherence to hydroxychloroquine and SLE flares, damage, and mortality in 660 patients from the SLICC Inception Cohort
Manuscript number (if known): ar-22-1545.R1

In the interest of transparency, we ask you to disclose all relationships/activities/interests listed below that are related to the content of your manuscript. "Related" means any relation with for-profit or not-for-profit third parties whose interests may be affected by the content of the manuscript. Disclosure represents a commitment to transparency and does not necessarily indicate a bias. If you are in doubt about whether to list a relationship/activity/interest, it is preferable that you do so.

The following questions apply to the author's relationships/activities/interests as they relate to the current manuscript only.

The author's relationships/activities/interests should be defined broadly. For example, if your manuscript pertains to the epidemiology of hypertension, you should declare all relationships with manufacturers of antihypertensive medication, even if that medication is not mentioned in the manuscript.

In item #1 below, report all support for the work reported in this manuscript without time limit. For all other items, the time frame for disclosure is the past 36 months.

		**Name all entities with whom you have this relationship or indicate none (add rows as needed)**	**Specifications/Comments (e.g., if payments were made to you or to your institution)**
**Time frame: Since the initial planning of the work**			
1	All support for the present manuscript (e.g., funding, provision of study materials, medical writing, article processing charges, etc.) **No time limit for this item.**	_X__None	
**Time frame: past 36 months**			
2	Grants or contracts from any entity (if not indicated in item #1 above).	_X__None	
3	Royalties or licenses	_X__None	
4	Consulting fees	_X__None	

5	Payment or honoraria for lectures, presentations, speakers bureaus, manuscript writing or educational events	_X__None	
6	Payment for expert testimony	_X__None	
7	Support for attending meetings and/or travel	_X__None	
8	Patents planned, issued or pending	_X__None	
9	Participation on a Data Safety Monitoring Board or Advisory Board	_X__None	
10	Leadership or fiduciary role in other board, society, committee or advocacy group, paid or unpaid	_X__None	
11	Stock or stock options	_X__None	
12	Receipt of equipment, materials, drugs, medical writing, gifts or other services	_X__None	
13	Other financial or non-financial interests	_X__None	

Please place an "X" next to the following statement to indicate your agreement:

_X__ I certify that I have answered every question and have not altered the wording of any of the questions on this form.

ICMJE DISCLOSURE FORM

Date: February 14th 2023
Your Name:___Anca Askanase_______________________
Manuscript Title : Association between severe non-adherence to hydroxychloroquine and SLE flares, damage, and mortality in 660 patients from the SLICC Inception Cohort
Manuscript number (if known): ar-22-1545.R1

In the interest of transparency, we ask you to disclose all relationships/activities/interests listed below that are related to the content of your manuscript. "Related" means any relation with for-profit or not-for-profit third parties whose interests may be affected by the content of the manuscript. Disclosure represents a commitment to transparency and does not necessarily indicate a bias. If you are in doubt about whether to list a relationship/activity/interest, it is preferable that you do so.

The following questions apply to the author's relationships/activities/interests as they relate to the current manuscript only.

The author's relationships/activities/interests should be defined broadly. For example, if your manuscript pertains to the epidemiology of hypertension, you should declare all relationships with manufacturers of antihypertensive medication, even if that medication is not mentioned in the manuscript.

In item #1 below, report all support for the work reported in this manuscript without time limit. For all other items, the time frame for disclosure is the past 36 months.

		**Name all entities with whom you have this relationship or indicate none (add rows as needed)**	**Specifications/Comments (e.g., if payments were made to you or to your institution)**
**Time frame: Since the initial planning of the work**			
1	All support for the present manuscript (e.g., funding, provision of study materials, medical writing, article processing charges, etc.) **No time limit for this item.**	__X__None	
**Time frame: past 36 months**			
2	Grants or contracts from any entity (if not indicated in item #1 above).	__X__None	
3	Royalties or licenses	__X__None	
4	Consulting fees	Abbvie, AstraZeneca, Aurinia, BMS, Celgene, Eli	•

		Lilly, Idorsia, Janssen, Genentech, GSK, Mallinckrodt, Pfizer and UCB	
5	Payment or honoraria for lectures, presentations, speakers bureaus, manuscript writing or educational events	__X__None	
6	Payment for expert testimony	__X__None	
7	Support for attending meetings and/or travel	__X__None	
8	Patents planned, issued or pending	__X__None	
9	Participation on a Data Safety Monitoring Board or Advisory Board	Amgen	
10	Leadership or fiduciary role in other board, society, committee or advocacy group, paid or unpaid	__X__None	
11	Stock or stock options	__X__None	
12	Receipt of equipment, materials, drugs, medical writing, gifts or other services	__X__None	
13	Other financial or non-financial interests	__X__None	

Please place an "X" next to the following statement to indicate your agreement:

_X__ I certify that I have answered every question and have not altered the wording of any of the questions on this form.

ICMJE DISCLOSURE FORM

Date: February 14th 2023
Your Name: Jill P. Buyon, MD
Manuscript Title : Association between severe non-adherence to hydroxychloroquine and SLE flares, damage, and mortality in 660 patients from the SLICC Inception Cohort
Manuscript number (if known): ar-22-1545.R1

In the interest of transparency, we ask you to disclose all relationships/activities/interests listed below that are related to the content of your manuscript. "Related" means any relation with for-profit or not-for-profit third parties whose interests may be affected by the content of the manuscript. Disclosure represents a commitment to transparency and does not necessarily indicate a bias. If you are in doubt about whether to list a relationship/activity/interest, it is preferable that you do so.

The following questions apply to the author's relationships/activities/interests as they relate to the current manuscript only.

The author's relationships/activities/interests should be defined broadly. For example, if your manuscript pertains to the epidemiology of hypertension, you should declare all relationships with manufacturers of antihypertensive medication, even if that medication is not mentioned in the manuscript.

In item #1 below, report all support for the work reported in this manuscript without time limit. For all other items, the time frame for disclosure is the past 36 months.

		**Name all entities with whom you have this relationship or indicate none (add rows as needed)**	**Specifications/Comments (e.g., if payments were made to you or to your institution)**
		**Time frame: Since the initial planning of the work**	
1	All support for the present manuscript (e.g., funding, provision of study materials, medical writing, article processing charges, etc.) **No time limit for this item.**	X None	
		**Time frame: past 36 months**	
2	Grants or contracts from any entity (if not indicated in item #1 above).	X None	
3	Royalties or licenses	X None	
4	Consulting fees	X None	

5	Payment or honoraria for lectures, presentations, speakers bureaus, manuscript writing or educational events	X None	
6	Payment for expert testimony	X None	
7	Support for attending meetings and/or travel	X None	
8	Patents planned, issued or pending	X None	
9	Participation on a Data Safety Monitoring Board or Advisory Board	X None	
10	Leadership or fiduciary role in other board, society, committee or advocacy group, paid or unpaid	X None	
11	Stock or stock options	X None	
12	Receipt of equipment, materials, drugs, medical writing, gifts or other services	X None	
13	Other financial or non-financial interests	X None	

Please place an "X" next to the following statement to indicate your agreement:

X I certify that I have answered every question and have not altered the wording of any of the questions on this form.

ICMJE DISCLOSURE FORM

Date: February 15th 2023
Your Name: MD PhD Nathalie COSTEDOAT-CHALUMEAU
Manuscript Title : Association between severe non-adherence to hydroxychloroquine and SLE flares, damage, and mortality in 660 patients from the SLICC Inception Cohort
Manuscript number (if known): ar-22-1545.R1

In the interest of transparency, we ask you to disclose all relationships/activities/interests listed below that are related to the content of your manuscript. "Related" means any relation with for-profit or not-for-profit third parties whose interests may be affected by the content of the manuscript. Disclosure represents a commitment to transparency and does not necessarily indicate a bias. If you are in doubt about whether to list a relationship/activity/interest, it is preferable that you do so.

The following questions apply to the author's relationships/activities/interests as they relate to the current manuscript only.

The author's relationships/activities/interests should be defined broadly. For example, if your manuscript pertains to the epidemiology of hypertension, you should declare all relationships with manufacturers of antihypertensive medication, even if that medication is not mentioned in the manuscript.

In item #1 below, report all support for the work reported in this manuscript without time limit. For all other items, the time frame for disclosure is the past 36 months.

		**Name all entities with whom you have this relationship or indicate none (add rows as needed)**	**Specifications/Comments (e.g., if payments were made to you or to your institution)**
**Time frame: Since the initial planning of the work**			
1	All support for the present manuscript (e.g., funding, provision of study materials, medical writing, article processing charges, etc.) **No time limit for this item.**	_X__None	
**Time frame: past 36 months**			
2	Grants or contracts from any entity (if not indicated in item #1 above).	_X__None	
3	Royalties or licenses	__X_None	
4	Consulting fees	__X_None	

5	Payment or honoraria for lectures, presentations, speakers bureaus, manuscript writing or educational events	__X__None	
6	Payment for expert testimony	__X__None	
7	Support for attending meetings and/or travel	__X__None	
8	Patents planned, issued or pending	__X__None	
9	Participation on a Data Safety Monitoring Board or Advisory Board	__X__None	
10	Leadership or fiduciary role in other board, society, committee or advocacy group, paid or unpaid	__X__None	
11	Stock or stock options	__X__None	
12	Receipt of equipment, materials, drugs, medical writing, gifts or other services	__X__None	
13	Other financial or non-financial interests	__X__None	

Please place an "X" next to the following statement to indicate your agreement:

__X_ I certify that I have answered every question and have not altered the wording of any of the questions on this form.
