# Supplementary material for: Association Between Severe Nonadherence to Hydroxychloroquine and Systemic Lupus Erythematosus Flares, Damage, and Mortality in 660 Patients From the SLICC Inception Cohort
Source: Arthritis Rheumatol. 2023 Nov 13;75(12):2195–206. doi: 10.1002/art.42645 (PMC10792124; doi:10.1002/art.42645)
Supplement: Supplementary file 2 — Appendix S1: Supporting Information [file ART-75-2195-s002.docx]

**SUPPLEMENTARY APPENDIX**

**Supplementary Figure S1. Flow chart**


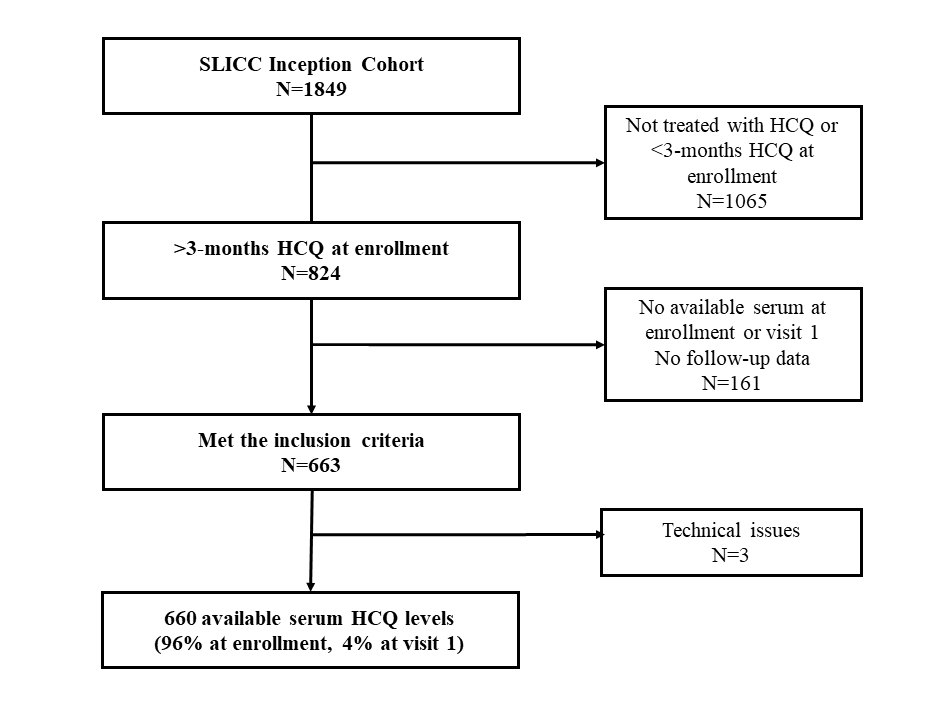


Abbreviations: SLICC: Systemic Lupus International Collaborating Clinics; HCQ: hydroxychloroquine;

**Supplementary Figure S2.** Histogram of the delay between hydroxychloroquine (HCQ) and azathioprine (AZA) start date in days, according to HCQ severe non-adherence. Negative values indicate that azathioprine began before HCQ.


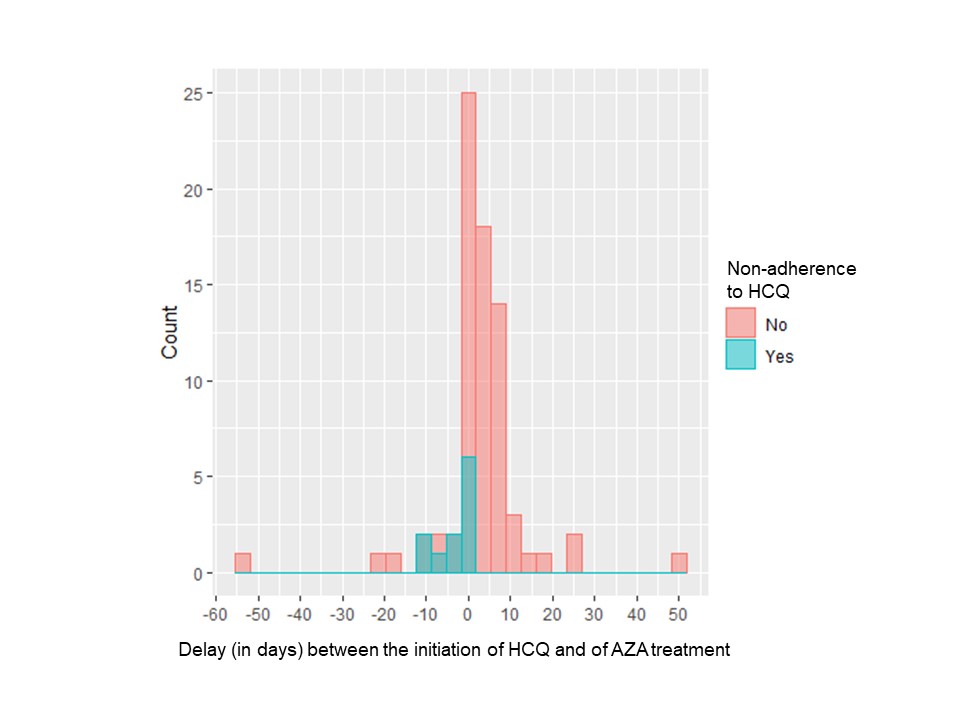


**Supplementary Table S1.** Comparisons between patients included in the study population meeting the inclusion criteria and excluded patients in the SLICC inception cohort (N=1,846). Patients meeting inclusion criteria and excluded due to technical issues were excluded from this table (N=3).

| **Characteristic** | **Overall SLICC cohort**  N = 1,846^1^ | **Study population**  N = 660^1^ | **Excluded population**  N = 1,186^1^ | **p-value**^2^ |
| --- | --- | --- | --- | --- |
| Sex |  |  |  | 0.4 |
| Female | 1,639 (89%) | 580 (88%) | 1,059 (89%) |  |
| Male | 207 (11%) | 80 (12%) | 127 (11%) |  |
| Black ethnicity | 308 (17%) | 107 (16%) | 201 (17%) | 0.7 |
| Age at SLE diagnosis | 32 (24, 43) | 34 (25, 44) | 31 (23, 42) | 0.002 |
| Months between SLE diagnosis and inclusion | 5.0 (2.0, 9.0) | 6.8 (3.3, 10.5) | 4.0 (1.0, 8.0) | <0.001 |
| Education level |  |  |  | <0.001 |
| Postsecondary | 1,060 (57%) | 413 (63%) | 647 (55%) |  |
| ≤High School | 786 (43%) | 247 (37%) | 539 (45%) |  |
| Cigarette smoking |  |  |  | 0.4 |
| Current or past smoker | 647 (35%) | 222 (34%) | 425 (36%) |  |
| Non-smoker | 1,198 (65%) | 437 (66%) | 761 (64%) |  |
| Unknown | 1 | 1 | 0 |  |
| Main clinical manifestations |  |  |  |  |
| Renal disease | 511 (28%) | 131 (20%) | 380 (32%) | <0.001 |
| Neurologic disorder | 89 (4.8%) | 18 (2.7%) | 71 (6.0%) | 0.002 |
| ^1^n (%); Median (IQR); | | | | |
| ^2^Pearson's Chi-squared test; Wilcoxon rank sum test;  Abbreviations: SLE: systemic lupus erythematosus | | | | |

**Supplementary Table S2.** Odds ratios (95% confidence intervals) for the risk of ≥4-point increase in SLEDAI-2000 in the year after the serum HCQ level measurement (N=660).

|  |  |  | **≥4-point increase in SLEDAI-2000 within one year** | | |
| --- | --- | --- | --- | --- | --- |
|  |  |  | N events (%) or mean (SD)  N=68 | Univariate | Multivariate |
|  | **Overall**  **N=660** |  |  | OR (95%CI) | OR (95%CI) |
| ***Demographic data and comorbidities*** | | |  |  |  |
| Age at serum sample, years, *mean (SD)* | 36.2 (13.5) |  | 33.8 (13.3) | 0.98 (0.96-1.00) | 0.99 (0.97-1.01) |
| Male sex | 80 (12.7) |  | 8 (11.8) | 0.96 (0.41-1.99) |  |
| Black race | 107 (16.2) |  | 21 (30.9) | 2.63 (1.47-4.57) | 2.42 (1.32-4.32) |
| Education level |  |  |  |  |  |
| Post-secondary | 247 (37.4) |  | 42 (61.8) | Reference | Reference |
| ≤High school | 413 (62.6) |  | 26 (38.2) | 1.04 (0.61-1.73) | 1.11 (0.64-1.90) |
| Cigarette smoking |  |  |  |  |  |
| Non-smoker | 437 (66.2) |  | 46 (68.7) | Reference |  |
| Current or past smoker | 222 (33.6) |  | 21 (31.3) | 1.24 (0.61-2.38) |  |
| Body mass index, kg/m²**,** *mean (SD)* | 25.8 (6.1) |  | 26.1 (6.0) | 1.01 (0.96-1.05) |  |
| ***SLEDAI-2000*,** *mean (SD)* | 4.84 (4.85) |  | 2.9 (2.8) | 0.87 (0.81-0.94) |  |
| ***Treatment*** | | |  |  |  |
| Corticosteroid | 438 (66.4) |  | 52 (76.5) | 1.73 (0.99-3.21) | 1.88 (1.02-3.62) |
| Azathioprine | 91 (13.8) |  | 14 (20.6) | 1.73 (0.89-3.19) | 1.44 (0.70-2.81) |
| Hydroxychloroquine |  |  |  |  |  |
| Severe HCQ non-adherence | 48 (7.3) |  | 11 (16.2) | 2.89 (1.34-5.82) | 3.19 (1.42-6.81) |
| Non-quantifiable serum levels | 28 (4.2) |  | 5 (7.4) | 1.96 (0.64-4.96) |  |

**Legends**: Results are expressed as N (%) for categorical variables and means (SD) for continuous variables. Following variables were included in the multivariate model: age, black race, education level (post-secondary; ≤High school), SLEDAI-2000, corticosteroids, azathioprine, and severe HCQ non-adherence.

Abbreviations: SD: standard deviation; OR: odds ratio; 95% CI: 95% confidence interval; kg/m²: kilograms per square meter; SLEDAI-2K: SLE disease activity index 2000; HCQ: hydroxychloroquine;

**Supplementary Table S3.** Odds ratios (95% confidence intervals) for the risk of new prescription of prednisone and/or another immunosuppressive drug in the year after the serum HCQ level measurement (N=660).

|  |  |  | **New prescription of prednisone or other immunosuppressant within one year** | | |
| --- | --- | --- | --- | --- | --- |
|  |  |  | N events (%) or mean (SD)  N=94 | Univariate | Multivariate |
|  | **Overall**  **N=660** |  |  | OR (95%CI) | OR (95%CI) |
| ***Demographic data and comorbidities*** | |  | |  |  |
| Age at serum sample, years**,** *mean (SD)* | 36.2 (13.5) |  | 34.6 (12.1) | 0.99 (0.97-1.01) | 0.99 (0.97-1.01) |
| Male sex | 80 (12.7) |  | 9 (9.6) | 0.74 (0.33-1.46) |  |
| Black race | 107 (16.2) |  | 20 (21.3) | 1.49 (0.85-2.52) | 1.46 (0.81-2.53) |
| Education level |  |  |  |  |  |
| Post-secondary | 247 (37.4) |  | 55 (58.5) | Reference | Reference |
| ≤High school | 413 (62.6) |  | 39 (41.5) | 1.22 (0.78-1.90) | 1.27 (0.80-2.01) |
| Cigarette smoking |  |  |  |  |  |
| Non-smoker | 437 (66.2) |  | 63 (67.0) | Reference |  |
| Current or past smoker | 222 (33.6) |  | 31 (33.0) | 0.96 (0.60-1.52) |  |
| Body mass index, kg/m²**,** *mean (SD)* | 25.8 (6.1) |  | 25.7 (5.5) | 1.00 (0.96-1.03) |  |
| ***SLEDAI-2000*,** *mean (SD)* | 4.8 (4.9) |  | 5.8 (5.6) | 1.04 (1.00-1.09) | 1.05 (1.00-1.09) |
| ***Treatment*** | |  | |  |  |
| Corticosteroid | 438 (66.4) |  | 57 (60.6) | 0.75 (0.48-1.18) | 0.57 (0.34-0.93) |
| Azathioprine | 91 (13.8) |  | 15 (16.0) | 1.22 (0.65-2.18) | 1.12 (0.57-2.11) |
| Hydroxychloroquine |  |  |  |  |  |
| Severe HCQ nonadherence | 48 (7.3) |  | 16 (17.0) | 3.42 (1.76-6.45) | 3.16 (1.59-6.07) |
| Non-quantifiable serum levels | 28 (4.2) |  | 8 (8.5) | 2.54 (1.02-5.75) |  |

**Legends**: Results are expressed as N (%) for categorical variables and means (SD) for continuous variables. New immunosuppressive agent included azathioprine, methotrexate, mycophenolate mofetil, rituximab, oral or IV cyclophosphamide. Following variables were included in the multivariate model: age, black race, education level (post-secondary; ≤High school), SLEDAI-2000, corticosteroids, azathioprine, and severe HCQ non-adherence.

Abbreviations: SD: standard deviation; OR: odds ratio; 95% CI: 95% confidence interval; kg/m²: kilograms per square meter; SLEDAI-2K: SLE disease activity index 2000; HCQ: hydroxychloroquine;

**Supplementary Table S4.** Odds ratios (95% confidence intervals) for the risk of new renal involvement in the year after serum HCQ level measurement (N=660).

|  |  |  | **New renal involvement within one year** | | |
| --- | --- | --- | --- | --- | --- |
|  |  |  | N events (%) or mean (SD)  N=71 | Univariate | Multivariate |
|  | **Overall** |  |  | OR (95%CI) | OR (95%CI) |
| ***Demographic data and comorbidities*** | | |  |  |  |
| Age at serum sample, years, *mean (SD)* | 36.2 (13.5) |  | 29.9 (10.3) | 0.95 (0.93-0.97) | 0.96 (0.94-0.98) |
| Male sex | 80 (12.7) |  | 11 (15.5) | 1.38 (0.66-2.66) |  |
| Black race | 107 (16.2) |  | 22 (31.0) | 2.66 (1.51-4.58) | 2.54 (1.37-4.61) |
| Education level |  |  |  |  |  |
| Post-secondary | 247 (37.4) |  | 43 (60.6) | - | - |
| ≤High school | 413 (62.6) |  | 28 (39.4) | 1.10 (0.66-1.81) | 0.83 (0.47-1.43) |
| Cigarette smoking |  |  |  |  |  |
| Non-smoker | 437 (66.2) |  | 49 (69.0) | - | - |
| Current or past smoker | 222 (33.6) |  | 22 (31.0) | 0.87 (0.50-1.46) | - |
| Body mass index, kg/m²**,** *mean (SD)* | 25.79 (6.06) |  | 25.8 (6.2) | 1.00 (0.96-1.04) |  |
| ***SLEDAI-2000*,** *mean (SD)* | 4.8 (4.9) |  | 8.7 (6.6) | 1.15 (1.10-1.20) | 1.13 (1.08-1.18) |
| ***Treatment*** | | |  |  |  |
| Corticosteroid | 438 (66.4) |  | 64 (90.1) | 5.26 (2.53-12.79) | 2.48 (1.10-6.36) |
| Azathioprine | 91 (13.8) |  | 15 (21.1) | 1.81 (0.95-3.28) | 1.19 (0.58-2.30) |
| Hydroxychloroquine |  |  |  |  |  |
| Severe HCQ non-adherence | 48 (7.3) |  | 9 (12.7) | 2.05 (0.89-4.25) | 1.41 (0.56-3.25) |
| Non-quantifiable serum levels | 28 (4.2) |  | 6 (8.5) | 2.38 (0.85-5.75) |  |

**Legends**: Results are expressed as N (%) for categorical variables and means (SD) for continuous variables. New renal involvement included active nephritis (defined by any of hematuria >5 red blood cells/hf excluding other causes; pyuria > 5 white blood cells/hpf, excluding infection; new or recent increase of >500 mg 24-hour protein; heme granular or RBC casts), a new nephrotic syndrome. Following variables were included in the multivariate model: age, black race, education level (post-secondary; ≤High school), SLEDAI-2000, corticosteroids, azathioprine, and severe HCQ non-adherence.

Abbreviations: SD: standard deviation; OR: odds ratio; 95% CI: 95% confidence interval; kg/m²: kilograms per square meter; SLEDAI-2K: SLE disease activity index 2000; HCQ: hydroxychloroquine;

**Supplementary Table S5.** Sensitivity analyses assessing the odds ratios (95% confidence interval) for the risk of SLE flare defined by the composite endpoint, ≥4-point increase in SLEDAI-2K, a new prescription of prednisone or immunosuppressant, and a new renal involvement within one year following time 0, according to alternate definitions of severe non-adherence (N=660)

| Alternate definitions of severe non-adherence to HCQ (regardless of the daily HCQ dose) | SLE flare | ≥4-point increase in SLEDAI-2K | New prednisone or immunosuppressant | New renal involvement |
| --- | --- | --- | --- | --- |
|  | OR (95%CI) | OR (95%CI) | OR (95%CI) | OR (95%CI) |
| **≤106 ng/mL threshold (N=58, 8/8%)** |  |  |  |  |
| Univariate analysis | 2.51 (1.45-4.34) | 2.24 (1.05-4.42) | 2.56 (1.34-4.69) | 1.60 (0.71-3.27) |
| Multivariate analysis* | 2.38 (1.34–4.22) | 2.65 (1.21-5.45) | 2.43 (1.27-4.48) | 1.14 (0.45-2.58 |
| **≤53 ng/mL threshold (N=42, 6.4%)** |  |  |  |  |
| Univariate analysis | 3.60 (1.91-6.89) | 3.02 (1.35-6.26) | 2.97 (1.44-5.85) | 2.07 (0.86-4.47) |
| Multivariate analysis* | 3.01 (1.55–5.94) | 3.47 (1.46–7.75) | 2.76 (1.30–5.58) | 1.26 (0.47-3.06) |

*Following variables were included in the multivariate model: age, black race, education level (post-secondary; ≤High school), SLEDAI-2K, corticosteroids, azathioprine, and severe HCQ non-adherence.

Abbreviations: SD: standard deviation; OR: odds ratio; 95% CI: 95% confidence interval; SLEDAI-2K: SLE disease activity index 2000; HCQ: hydroxychloroquine.

**Supplementary Table S6.** Comparisons of damage among patients with ≥1-point increase of SLICC/ACR Damage Index within 5 years after time zero, by hydroxychloroquine severe non-adherence status (N=167)

| **Patients' damage** | **Overall (n=167)** | **Severe non-adherence to hydroxychloroquine** | | **p-value** |
| --- | --- | --- | --- | --- |
|  |  | **No (n=152)** | **Yes (n=15)** |  |
| **Cataract*** | 23 (13.8) | 21 (13.8) | 2 (13.3) | 1 |
| **Retinal change or optic atrophy*** | 7 (4.2) | 7 (4.6) | 0 (0.0) | 0.862 |
| Cognitive impairment or psychosis | 12 (7.2) | 11 (7.2) | 1 (6.7) | 1 |
| Seizures requiring therapy for >6 months | 3 (1.8) | 3 (2.0) | 0 (0.0) | 1 |
| Stroke |  |  |  | 0.499 |
| Single episode | 11 (6.6) | 11 (7.2) | 0 (0.0) |  |
| Two or more episodes | 2 (1.2) | 2 (1.3) | 0 (0.0) |  |
| Cranial or peripheral neuropathy | 11 (6.6) | 9 (5.9) | 2 (13.3) | 0.576 |
| Transverse myelitis | 5 (3.0) | 4 (2.6) | 1 (6.7) | 0.936 |
| Estimated or measured GFR <50% for >6 months | 7 (4.2) | 7 (4.6) | 0 (0.0) | 0.862 |
| Proteinuria ≥3 5 g/24 hours for >6 months | 13 (7.8) | 11 (7.2) | 2 (13.3) | 0.737 |
| End-stage renal disease | 3 (1.8) | 2 (1.3) | 1 (6.7) | 0.316 |
| Pulmonary hypertension | 6 (3.6) | 5 (3.3) | 1 (6.7) | 1 |
| Pulmonary fibrosis | 6 (3.6) | 6 (3.9) | 0 (0.0) | 0.955 |
| Shrinking Lung | 1 (0.6) | 1 (0.7) | 0 (0.0) | 1 |
| Pleural fibrosis | 1 (0.6) | 1 (0.7) | 0 (0.0) | 1 |
| Pulmonary infarction | 2 (1.2) | 2 (1.3) | 0 (0.0) | 1 |
| Angina or coronary artery bypass | 4 (2.4) | 4 (2.6) | 0 (0.0) | 1 |
| Myocardial infarction |  |  |  | 0.775 |
| Single episode | 2 (1.2) | 2 (1.3) | 0 (0.0) |  |
| Two or more episodes | 3 (1.8) | 3 (2.0) | 0 (0.0) |  |
| Cardiomyopathy | 4 (2.4) | 4 (2.6) | 0 (0.0) | 1 |
| Valvular disease | 3 (1.8) | 2 (1.3) | 1 (6.7) | 0.639 |
| Pericarditis | 4 (2.4) | 3 (2.0) | 1 (6.7) | 0.803 |
| Claudication | 1 (0.6) | 1 (0.7) | 0 (0.0) | 1 |
| Minor tissue loss from peripheral arterial disease | 6 (3.6) | 5 (3.3) | 1 (6.7) | 1 |
| Significant tissue loss from peripheral arterial disease | 1 (0.6) | 1 (0.7) | 0 (0.0) | 1 |
| Venous thrombosis with swelling, ulceration or venous stasis | 9 (5.4) | 6 (3.9) | 3 (20.0) | 0.043 |
| Infarction or resection of bowel (below duodenum), Spleen, liver or gallbladder | 8 (4.8) | 7 (4.6) | 1 (6.7) | 1 |
| Mesenteric insufficiency | 0 (0) | 0 (0) | 0 (0) | NA |
| Chronic peritonitis | 0 (0) | 0 (0) | 0 (0) | NA |
| Stricture or upper gastrointestinal tract surgery | 1 (0.6) | 1 (0.7) | 0 (0.0) | 1 |
| Pancreatic insufficiency requiring enzyme replacement or with pseudocyst | 1 (0.6) | 1 (0.7) | 0 (0.0) | 1 |
| **Muscle atrophy or weakness*** | 8 (4.8) | 8 (5.3) | 0 (0.0) | 0.782 |
| Deforming or erosive arthritis | 16 (9.6) | 15 (9.9) | 1 (6.7) | 1 |
| **Osteoporosis with fracture or vertebral collapse*** | 12 (7.2) | 12 (7.9) | 0 (0.0) | 0.499 |
| **Avascular necrosis*** |  |  |  | 0.534 |
| Single episode | 5 (3.0) | 4 (2.6) | 1 (6.7) |  |
| Two or more episodes | 6 (3.6) | 5 (3.3) | 1 (6.7) |  |
| Osteomyelitis | 1 (0.6) | 1 (0.7) | 0 (0.0) | 1 |
| Ruptured tendons | 0 (0) | 0 (0) | 0 (0) | NA |
| Scarring chronic alopecia | 22 (13.2) | 20 (13.2) | 2 (13.3) | 1 |
| Extensive scarring of panniculum other than scalp and pulp space | 9 (5.4) | 7 (4.6) | 2 (13.3) | 0.407 |
| Skin ulceration (excluding thrombosis) | 3 (1.8) | 3 (2.0) | 0 (0.0) | 1 |
| **Premature gonadal failure*** | 4 (2.4) | 4 (2.6) | 0 (0.0) | 1 |
| **Diabetes requiring therapy*** | 5 (3.0) | 5 (3.3) | 0 (0.0) | 1 |
| Malignancy | 13 (7.8) | 12 (7.9) | 1 (6.7) | 1 |
| **Patients with treatment related damage** | 53 (31.7) | 51 (33.6) | 2 (13.3) | 0.19 |
| **Patients with other damage** | 138 (82.6) | 123 (80.9) | 15 (100) | 0.133 |

**Legends:** *Damage included in treatment-related damage. Abbreviations: GFR: glomerular filtration rate; NA: not applicable. Patients could have had numerous forms of damage.
